# Antibody production and tolerance to the α-gal epitope as models for understanding and preventing the immune response to incompatible ABO carbohydrate antigens and for α-gal therapies

**DOI:** 10.3389/fmolb.2023.1209974

**Published:** 2023-06-28

**Authors:** Uri Galili

**Affiliations:** Department of Medicine, Rush University Medical College, Chicago, IL, United States

**Keywords:** ABO-incompatible antigens, immune tolerance, alpha-gal epitope, anti-Gal, alpha-gal therapies, alpha-gal nanoparticles, immune accommodation

## Abstract

This review describes the significance of the α-gal epitope (Galα-3Galβ1-4GlcNAc-R) as the core of human blood-group A and B antigens (A and B antigens), determines in mouse models the principles underlying the immune response to these antigens, and suggests future strategies for the induction of immune tolerance to incompatible A and B antigens in human allografts. Carbohydrate antigens, such as ABO antigens and the α-gal epitope, differ from protein antigens in that they do not interact with T cells, but B cells interacting with them require T-cell help for their activation. The α-gal epitope is the core of both A and B antigens and is the ligand of the natural anti-Gal antibody, which is abundant in all humans. In A and O individuals, anti-Gal clones (called anti-Gal/B) comprise >85% of the so-called anti-B activity and bind to the B antigen in facets that do not include fucose-linked α1–2 to the core α-gal. As many as 1% of B cells are anti-Gal B cells. Activation of quiescent anti-Gal B cells upon exposure to α-gal epitopes on xenografts and some protozoa can increase the titer of anti-Gal by 100-fold. α1,3-Galactosyltransferase knockout (GT-KO) mice lack α-gal epitopes and can produce anti-Gal. These mice simulate human recipients of ABO-incompatible human allografts. Exposure for 2–4 weeks of naïve and memory mouse anti-Gal B cells to α-gal epitopes in the heterotopically grafted wild-type (WT) mouse heart results in the elimination of these cells and immune tolerance to this epitope. Shorter exposures of 7 days of anti-Gal B cells to α-gal epitopes in the WT heart result in the production of accommodating anti-Gal antibodies that bind to α-gal epitopes but do not lyse cells or reject the graft. Tolerance to α-gal epitopes due to the elimination of naïve and memory anti-Gal B cells can be further induced by 2 weeks *in vivo* exposure to WT lymphocytes or autologous lymphocytes engineered to present α-gal epitopes by transduction of the α1,3-galactosyltransferase gene. These mouse studies suggest that autologous human lymphocytes similarly engineered to present the A or B antigen may induce corresponding tolerance in recipients of ABO-incompatible allografts. The review further summarizes experimental works demonstrating the efficacy of α-gal therapies in amplifying anti-viral and anti-tumor immune-protection and regeneration of injured tissues.

## Introduction

The objectives of this review are to describe the immune significance of the α-gal epitope as the core of human blood-group A and B antigens (referred to as A and B antigens). The review further determines the principles underlying the immune response to these carbohydrate antigens by studying the anti-Gal immune response to α-gal epitopes in an experimental mouse model. The review also suggests a strategy for the induction of immune tolerance to A and B antigens as incompatible antigens in allografts based on studies in the experimental model and summarizes the experimental studies that suggest harnessing anti-Gal/α-gal epitope interaction for several α-gal therapies in humans.

The immune response to carbohydrate antigens differs from that to protein antigens and is less understood than the latter. These differences include the following: 1) anti-protein and anti-peptide antibodies are usually produced following exposure of the immune system to protein antigens, such as viral infections. In contrast, >100 anti-carbohydrate antibodies in humans are continuously produced throughout life as “natural antibodies” (Wiener, 1951; Springer, 1971; Ochsenbein et al., 1999; Blixt et al., 2004; Bovin, 2013; Stowell et al., 2014) against a wide range of carbohydrate antigens presented by ∼400 different bacterial strains that comprise the human gastrointestinal (GI) flora (Hooper and Macpherson, 2010). These immunizing bacteria comprise ∼25% of the fecal material (Gerritsen et al., 2011). Among the most known natural anti-carbohydrate antibodies are anti-blood-group A (anti-A), anti-blood-group B (anti-B) (Springer, 1971; Watkins, 1980), anti-Gal (Galili et al., 1984; McMorrow et al., 1997a; Parker et al., 1999; Galili, 2013a), anti-Forssman (Young et al., 1979; Kijimoto-Ochiai et al., 1981), and anti-N-glycolyl neuraminic acid (anti-Neu5Gc) antibodies (Merrick et al., 1978; Zhu and Hurst, 2002; Nguyen et al., 2005; Padler-Karavani et al., 2008). 2) A second important difference is that immunogenic protein antigens can activate both cytotoxic and helper T cells. However, with very few exceptions, most immunogenic carbohydrate antigens, including mammalian cell surface carbohydrate antigens, can activate B cells producing the corresponding antibodies but cannot activate T cells (Ishioka et al., 1992; Speir et al., 1999; Avci et al., 2013). Nevertheless, T-cell help is required for the isotype switch from IgM to IgG or IgA production and is provided by immunogenic proteins, which may be linked to the carbohydrate antigen or are administered together with the carbohydrate antigen. In the absence of T-cell help, the produced anti-carbohydrate antibodies are usually of the IgM class.

Much information on the human immune response to carbohydrate antigens has been obtained from patients grafted with kidney allografts presenting the incompatible A or B antigen. It was well established that transplantation of an A or B kidney into O recipient, B kidney into A recipient, and A kidney into B recipient led to rapid (hyperacute) rejection of the allograft. This rejection results from binding the recipient’s anti-blood-group antibody to the corresponding incompatible A or B antigen presented on the allograft endothelial cells, followed by the activation of the complement system by this antigen/antibody interaction. This complement activation results in complement-mediated lysis of the endothelial cells, which causes the occlusion of blood vessels, the collapse of the vascular bed, and graft rejection (Starzl et al., 1964). Studies initiated in the 1980s found that in many ABO-incompatible kidney-transplanted patients, in whom the anti-blood-group antibodies were removed by plasmapheresis, were splenectomized and received an immunosuppressive protocol for preventing anti-HLA-mediated rejection, their allografts further survived for years (Alexandre et al., 1987; Bannett et al., 1987; Chopek et al., 1987). Analysis of antibody production against the incompatible A or B antigen of the graft revealed, in some patients, the production of antibodies that bound to the incompatible antigen but did not cause complement-mediated lysis of the allografts (Latinne et al., 1989; Park et al., 2003; Garcia de Mattos Barbosa et al., 2018). This phenomenon was called “accommodation,” and these unique antibodies have been referred to as “accommodating” antibodies (Platt and Cascalho, 2023). In a proportion of the patients who did not reject the allograft, no antibody production against the incompatible blood-group antigen was detected, implying the occurrence of immune tolerance against that antigen (Tydén et al., 2003; Genberg et al., 2007; Holgersson et al., 2014). Similarly, infants who were transplanted with an ABO-incompatible heart were found not to reject the allograft and to develop immune tolerance or accommodation to the incompatible carbohydrate antigen (West et al., 2001; Urschel et al., 2013; Urschel and West, 2016).

**FIGURE 1:**
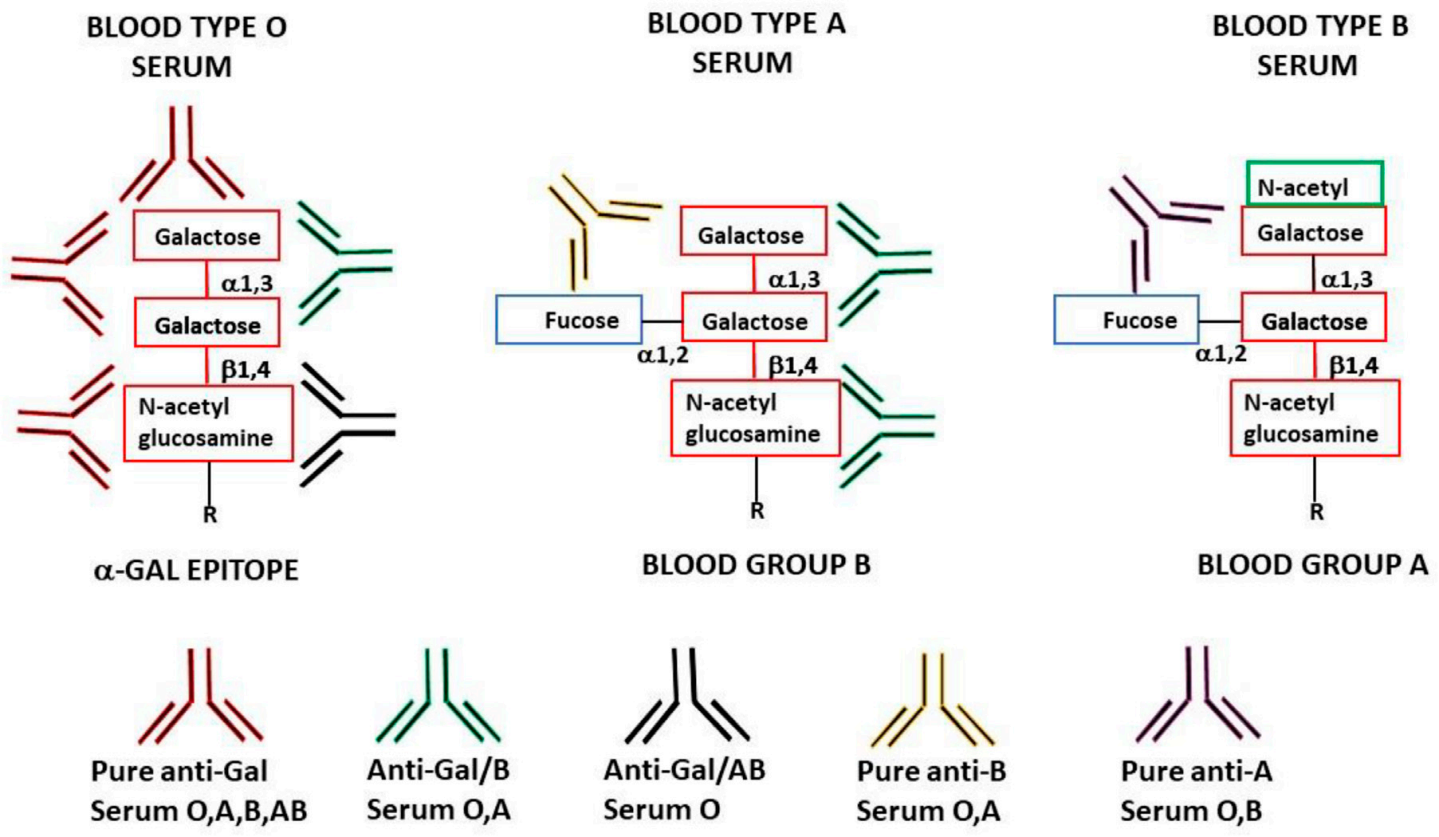
Schematic representation of anti-Gal antibody specificities in blood-type O, A, and B individuals. “Pure” anti-Gal (red antibody) is produced in all humans and binds to α-gal epitopes. These epitopes are absent in humans (except as the core of blood-group A and B antigens) but are synthesized in non-primate mammals, lemurs, and New World monkeys. Anti-Gal/B (green antibody) (comprises >85% of the total anti-B activity) is produced in blood-type O and A individuals and binds to the α-gal epitope and the α-gal core in blood-group B. Anti-Gal/AB (black antibody) is produced in blood-type O individuals and binds to the α-gal epitope and the α-gal core in blood-groups A and B. This antibody is present in small amounts in healthy individuals but may increase in O recipients of incompatible blood-group A allograft. “Pure” anti-B antibody (orange antibody) is produced in O and A individuals (comprises <15% of the total anti-B activity) and binds only to blood-group B red cells. “Pure” anti-A antibody (brown antibody) is produced in O and B individuals (comprises >97% of the total anti-A activity) and binds only to blood-group A red cells.

Research for understanding the immune response to incompatible carbohydrate antigens has been difficult because there has not been an appropriate experimental animal model in which some individuals lack a particular carbohydrate antigen and others synthesize that antigen and thus can serve as donors of a graft presenting an incompatible carbohydrate antigen. This limitation was overcome by studies on the anti-Gal antibody production against the α-gal epitope in α1,3-galactosyltransferase knockout (GT-KO) mice that lack α-gal epitopes (Thall et al., 1995; Tearle et al., 1996). Wild-type (WT) mice, like other non-primate mammals, synthesize the α-gal epitope with the structure Galα1-3Galβ1-4GlcNAc-R (Figure 1) through the glycosylation enzyme α1,3-galactosyltransferase (α1,3-GT), and present many of these epitopes on their cells. In contrast, GT-KO mice lack the α-gal epitope and can produce anti-Gal antibodies against this epitope (LaTemple and Galili, 1998; Tanemura et al., 2000a). The first part of this review describes the significance of the natural anti-Gal antibody (∼1% of human immunoglobulins) as an antibody model because it comprises much of the anti-B antibody activity and some of the anti-A activity. The significance of the α-gal epitope as an incompatible carbohydrate-antigen model emerges from the fact that it is the core of blood-group A and B carbohydrate structures (Figure 1). The second part discusses the principles of the immune response against incompatible carbohydrate antigens as understood from studies in anti-Gal-producing GT-KO mice and the relevance of these findings for the understanding and manipulation of the immune response in humans by tolerance induction to incompatible A and B antigens. The third part summarizes studies in GT-KO mice that demonstrate the efficacy of anti-Gal/α-gal epitope interaction in mediating α-gal therapies in various disciplines, including the amplification of immunogenicity of inactivated whole virus vaccines, *in situ* conversion of tumors into vaccines that elicit a protective immune response against autologous tumor antigens, and acceleration of wound healing and prevention of scar formation in skin and myocardial injuries.

## Anti-Gal immune response in humans

### Natural anti-Gal response

The natural anti-Gal antibody is one of the most abundant antibodies in all humans, and it is produced against environmental antigens, primarily α-gal-like epitopes present on the walls of the normal GI bacteria (Galili et al., 1988a; Mañez et al., 2001; Posekany et al., 2002). In fetal and newborn blood, anti-Gal is found as maternal IgG (Galili et al., 1984; Minanov et al., 1997; Doenz et al., 2000; Hamanova et al., 2015), whereas in children and adults, it is found as IgG, IgM, and IgA classes (Hamadeh et al., 1995; Hamanova et al., 2015). Maternal IgG reaches its lowest level at the age of 3–6 months; then, the infant starts producing its own anti-Gal induced by the bacterial flora established in the GI tract. In elderly individuals, anti-Gal titers are approximately half those in young individuals (Wang et al., 1995a).

The natural ligand of anti-Gal is the α-gal epitope with the structure Galα1-3Galβ1-4GlcNAc (Figure 1) (Galili et al., 1985), which is abundantly synthesized by α1,3-galactosyltransferase (α1,3-GT) on carbohydrate chains (glycans) of non-primate mammals, lemurs, and New World monkeys (monkeys of South America) (Galili et al., 1987a; Galili et al., 1988b; Oriol et al., 1999). In contrast, Old World monkeys (monkeys of Asia and Africa), apes, and humans all lack α-gal epitopes because of the evolutionary inactivation of the α1,3-GT gene (*GGTA1*) 20–30 million years ago (Galili and Swanson, 1991; Galili, 2023) and produce the natural anti-Gal antibody (Galili et al., 1987a; Teranishi et al., 2002). Anti-Gal has been of particular interest in the field of xenotransplantation, in which porcine organs, such as the kidney and heart, are studied as future xenografts in patients due to the paucity of such allograft organs for transplantation. The binding of this antibody to α-gal epitopes on endothelial cells in porcine xenografts transplanted into Old World monkeys or humans results in rapid (hyperacute) rejection of the xenograft due to anti-Gal mediated destruction and collapse of the vascular bed of the graft (Cooper et al., 1993; Galili, 1993; Sandrin et al., 1993; Collins et al., 1995). An initial step in developing methods for the future use of porcine xenografts has been the prevention of α-gal epitope synthesis in transgenic porcine by disruption of the α1,3-GT gene *GGTA1* (Lai et al., 2002; Phelps et al., 2003).

### Elicited anti-Gal response

**FIGURE 2:**
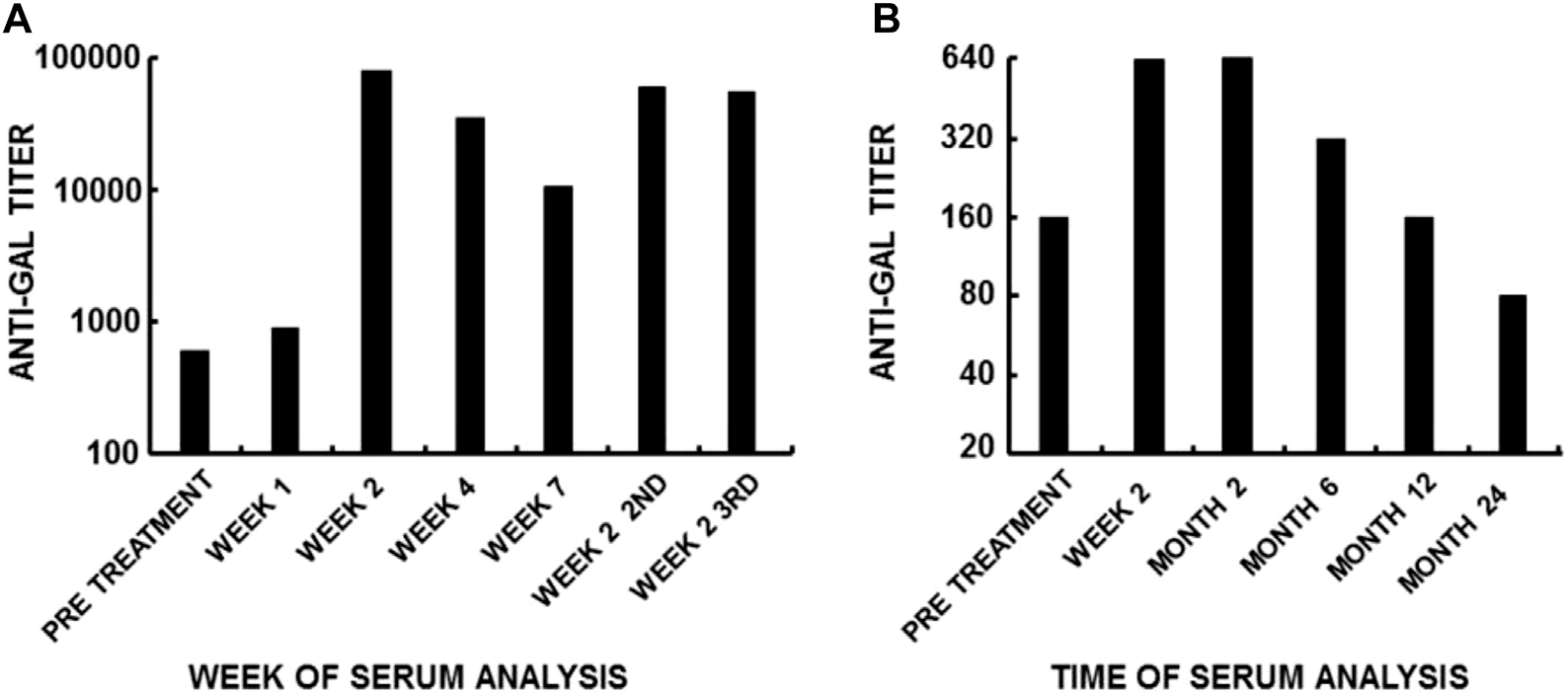
Anti-Gal immune response to α-gal epitopes on mammalian cells. Anti-Gal titers are presented as reciprocals of a serum dilution yielding half the maximum binding in ELISA using synthetic α-gal epitopes linked to BSA as solid-phase antigens. **(A)** Anti-Gal titers following three intraperitoneal infusions of 6 × 10^9^ 3T3-derived packaging mouse fibroblasts containing a replication-defective virus as part of a gene therapy experiment [modified from Galili et al. (2001)]. Note the >100-fold increase in anti-Gal titer within 14 days after infusion, the 10-fold decrease in Week 7, and the increase after the second and third infusions performed 7 weeks apart. **(B)** Increase in anti-Gal titer in a representative patient with a ruptured anterior cruciate ligament (ACL) who was implanted with a porcine patellar tendon enzymatically treated to remove α-gal epitopes from the tendon and then partially crosslinked by glutaraldehyde. Cell membranes presenting α-gal epitopes continuously leached out of the remodeled bone plugs attached to the tendon. The cells presenting α-gal epitopes within the bone cavities retained α-gal epitopes because the processing enzyme did not reach the bone cavities for eliminating these epitopes [modified from Stone et al. (2007)]. Note that anti-Gal activity remained elevated for several months and subsequently decreased as a result of the remodeling of the porcine bone into autologous human bone. From “Galili U. book “The natural anti-Gal antibody as foe turned friend in medicine.” Academic Press/Elsevier, London, 2018, with permission. pp. 13–16.

As many as 1% of circulating B cells in humans are quiescent B cells capable of producing anti-Gal, as shown by *in vitro* analysis of anti-Gal production among B cells immortalized by Epstein–Barr virus (EBV) (Galili et al., 1993). These quiescent anti-Gal B cells undergo robust activation following the encounter of α-gal epitopes on xenografts. Administration of mouse xenograft 3T3 cells presenting α-gal epitopes into patients (as part of experimental gene therapy studies) induces extensive activation of these B cells, resulting in a ∼100-fold increase in the titer of anti-Gal within 14 days (Galili et al., 2001). This increase is the result of a ∼10-fold increase in the number of anti-Gal-producing B cells within the first week, followed by an additional 10-fold increase in the affinity of the antibody in the second week due to affinity maturation by somatic mutations among clones of B cells producing anti-Gal. The immunizing cells are rapidly destroyed by anti-Gal, and the half-life of this elicited anti-Gal IgG is ∼3 weeks as that of other IgG molecules. Re-administration of mouse xenograft 3T3 cells after 7 weeks results in repeated production of anti-Gal at its peak of ∼100-fold the natural level and a similar peak following a third administration (Figure 2A). These findings imply that there is a regulatory mechanism that prevents the production of anti-carbohydrate antibodies above a maximum level. In patients implanted with processed porcine tendon, elicited anti-Gal IgG production reaches within 2–4 weeks a plateau that lasts for ∼4 months. This anti-Gal production is much higher than the natural level (Figure 2B) due to the continuous release of bone marrow and red cell membranes presenting α-gal epitopes from anchoring bone blocks (Stone et al., 2007). Once the porcine bone blocks are remodeled into autologous human bone, the anti-Gal level returns to its natural level. No toxic or other detrimental effects were caused by the prolonged high anti-Gal activity in the implanted patients. Activation of anti-Gal B cells for production of elicited anti-Gal was also observed in humans infected with protozoa presenting α-gal-like epitopes, such as *Trypanosoma* (Towbin et al., 1987; Milani and Travassos, 1988; Avila et al., 1989; Almeida et al., 1991; Almeida et al., 1994) and *Leishmania* (Avila et al., 1989). Elevated anti-Gal activity was shown to induce effective complement-mediated cytolysis of *Trypanosoma* (Milani and Travassos, 1988; Almeida et al., 1991; Almeida et al., 1994).

The elicited anti-Gal production due to the activation of anti-Gal B cells by xenograft α-gal epitopes is potent enough to overcome the immune suppression used for the prevention of kidney allograft rejection. This was shown in diabetic patients who were transplanted with kidney allograft and received during that procedure fetal porcine islet cells within the allograft subcapsular space or via the portal vein (Groth et al., 1994). The patients were treated with standard immunosuppression protocols that prevent T-cell mediated rejection due to the immune reaction against the HLA of allografts. Despite the successful prevention of kidney allograft rejection in these patients, antibody titers increased by 8–64-fold in IgG, IgM, and IgA anti-Gal, as well as the affinity of this antibody (Galili et al., 1995). These findings also reflect a robust anti-Gal response against the α-gal epitope on the porcine islet cells in patients under immune suppression treatments that are potent enough to prevent allograft rejection.

### Anti-Gal comprises most of the anti-blood-group B antibody activity

Human anti-Gal comprises multiple clones that bind to various “facets” of the α-gal epitope. The polyclonality of this antibody was suggested by the multiple pI values of anti-Gal, ranging from 4.0 to 8.5, as observed in isoelectric focusing (Galili et al., 1984). This polyclonality of the natural anti-Gal in humans was further confirmed by the observation that 8 out of 9 human anti-Gal-producing B cells immortalized by EBV transformation displayed the use of several VH3 heavy chain genes. These genes included various D and J genes, and comparisons with the corresponding germ-line genes demonstrated a number of replacement and silent mutations within the complementarity-determined regions (CDRs) (Wang et al., 1995b). These somatic mutations may provide a pool of variants that are available for affinity maturation, as described in the aforementioned section on the elicited anti-Gal response.

**FIGURE 3:**
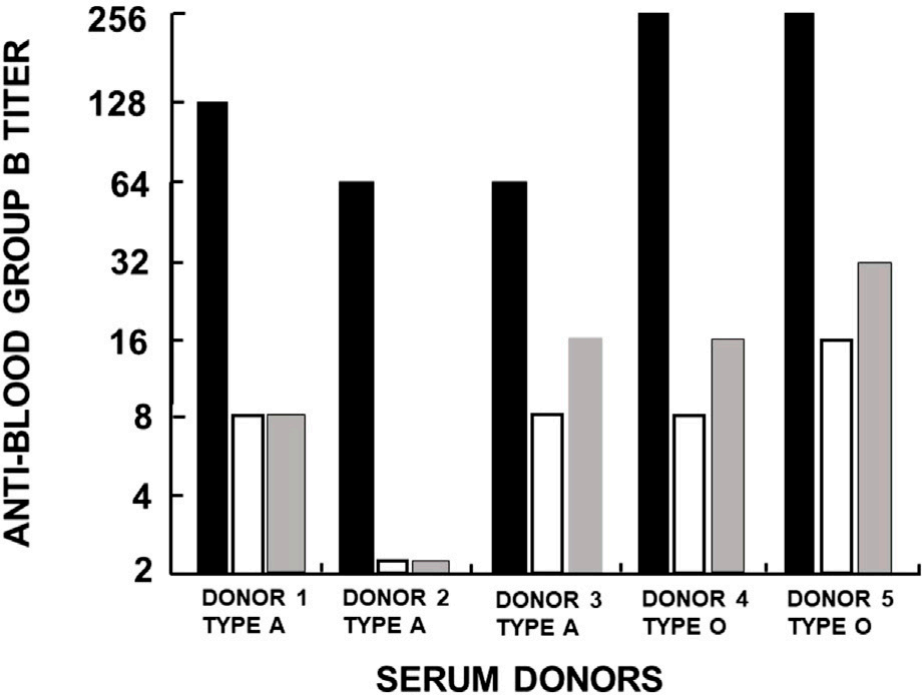
Analysis of anti-Gal/B antibodies produced in healthy individuals with blood types A and O. The activity (titer representing reciprocal serum dilution) of anti-Gal/B antibodies was derived from the decrease in agglutination of blood-group B red cells following adsorption of sera on an equal volume of packed rabbit red cells presenting natural α-gal epitopes (open columns) or on synthetic α-gal epitopes on silica beads (gray columns). Original anti-B activity in sera is presented as closed columns. The changes in B red cell agglutination after adsorption indicate that anti-Gal/B antibodies comprise ∼85%–95% of the so-called anti-blood-group B antibodies. Reproduced from Galili U. book “The natural anti-Gal antibody as foe turned friend in medicine,” Academic Press/Elsevier, London, 2018, with permission, pp. 50–52, and based on Galili et al. (1987b).

Another manifestation of anti-Gal polyclonality is that many of the anti-Gal clones comprise most of the so-called anti-blood-group B (anti-B) antibody activity, in addition to their binding to the α-gal epitope (Galili et al., 1987b; McMorrow et al., 1997b). As shown in Figure 1, the α-gal epitope is the core structure of blood-group B antigen, which differs from the α-gal epitope only by a fucose α1-2 linked to the penultimate galactose. Anti-Gal clones in blood-group B and AB individuals bind only to the α-gal epitope (called “pure” anti-Gal clones) because of the immune-tolerance mechanism, which prevents the appearance of any antibody to the core of B antigen. This inability to bind to the B antigen is caused by the fucose α1-2 linked to the penultimate galactose (Galili et al., 1987b). However, in A and O individuals, some of the anti-Gal clones bind to the α-gal epitope and blood-group B antigen (called anti-Gal/B antibodies) (Galili et al., 1987b; Galili et al., 2002). In fact, anti-Gal/B comprises >85% of anti-B antibodies in A and O sera and can be removed by adsorption on rabbit red cells and when A or O sera are passed through a column of synthetic α-gal epitopes (Figure 3). Moreover, the eluted antibodies from such a column demonstrate binding to α-gal epitopes and the B antigen (Galili et al., 1987b). These anti-Gal/B antibody clones comprise ∼50% of anti-Gal antibody activity in A and O individuals. Evidently, no anti-Gal/B clones are found in B or AB serum, where 100% of anti-Gal is “pure.” The remaining anti-B antibody clones (called “pure” anti-B), which do not bind to the α-gal epitope, require the presence of the Fucα1-2 linked to the penultimate galactose to bind to the B antigen (Figure 1). Analysis of anti-Gal clones in O individuals could also demonstrate the production of anti-Gal/AB activity, that is, anti-Gal clones capable of binding to A and B antigens (Figure 1) (Galili et al., 1987b). Anti-Gal/AB activity is severalfold lower than that of anti-Gal/B activity. However, as described in the following section, anti-Gal/AB activity can be markedly elevated in O recipients of an allograft presenting A or B antigen (Galili et al., 2002).

A crystallization study with the monoclonal anti-Gal antibody M86 immunocomplexed with the Galα1-3Gal portion of the α-gal epitope demonstrated a groove in the binding site of this antibody, in which the Galα1-3Gal disaccharide binds via hydrogen bonds to the antibody (Langley et al., 2022). In view of this study, it is possible that the groove shape in the binding site of pure anti-Gal antibody clones differs from that of anti-Gal/B in that in the latter, the binding is not affected by the fucose-linked α1-2 to the penultimate galactose, as schematically shown in Figure 1 (Galili et al., 1987b).

### Elicited anti-Gal/B and anti-Gal/AB production in patients transplanted with ABO-incompatible kidney allografts

The stimulation for the production of anti-Gal/B and anti-Gal/AB by the human B or A antigens could be evaluated in patients transplanted with ABO-incompatible kidney allografts between 1989 and 1999 (Ishida et al., 2000). In order to minimize the risk of rejection of the ABO allografts, the patients underwent plasmapheresis and immunoadsorption on columns for the removal of the natural anti-A and anti-B antibodies. The patients further received an immunosuppressive treatment for T-cell suppression. Most of the patients were also splenectomized during the course of the grafting surgery. The allograft (received from family relative donors) survival rate was 76% after 1 year and 73% after 5 years (Ishida et al., 2000).

A total of 12 patients rejected the allograft, and their sera were analyzed for anti-A, anti-B, anti-Gal/B, and anti-Gal/AB (Galili et al., 2002). Although no changes in the activity of these antibodies were observed in nine of these patients, three patients displayed increased activities of some of these antibodies. These changes were analyzed to shed light on some of the aspects of the elicited antibody response against ABO mismatched antigens on human allografts. In that study, blood type O patient #1, transplanted with B kidney allograft, displayed after 7 weeks a marked increase in anti-A, anti-B, and anti-Gal despite the absence of α-gal epitopes in the human kidney allograft. Serum adsorption on B or A red cells resulted in the removal of 50% and 30% of anti-Gal activity, respectively. These findings implied that the incompatible B antigen stimulated the expansion of anti-Gal/B and anti-Gal/AB clones in addition to pure anti-B clones.

An increase in anti-A, anti-B, and anti-Gal antibody activities was also observed in O patient #2, transplanted with blood-group A kidney allograft (Galili et al., 2002). The titers of these antibodies in the serum sample obtained 10 weeks after transplantation were markedly higher than those obtained before transplantation. Adsorption of Week 10 serum with rabbit red cells (i.e., removal of anti-Gal) decreased anti-A activity by 50% and anti-B activity by 100%. These observations implied that the core α-gal epitope within blood-group A of the kidney allograft induced the immune system of the recipient to produce anti-Gal/AB that comprised half of the overall elicited anti-A activity. In addition, 100% of the so-called anti**-**B activity produced by this patient was, in fact, that of anti-Gal/B antibody clones.

Blood type A patient #3 was grafted with an AB kidney allograft. After 11 days, the serum displayed very high anti-B activity that caused graft rejection. Removal of anti-Gal by adsorption of the serum on rabbit red cells indicated that anti-Gal/B comprised ∼70% of the measured anti-B activity. The observations in these recipients of ABO-incompatible kidney allografts (Galili et al., 2002) imply that much of the elicited antibody response against incompatible blood-group A and B in allografts involves anti-Gal/B- and anti-Gal/AB-producing cells activated by the facets of core α-gal epitopes, which do not include the facet of Fucα1-2 linked to the penultimate galactose (Figure 1). In addition, there are antibody-producing cell clones activated by the facets of A and B antigens that include the Fucα1-2 linked to the penultimate galactose (“pure” anti-A and anti-B antibodies). When produced in recipients of the incompatible A or B allograft, anti-Gal/B and anti-Gal/AB, and “pure” anti-A and anti-B antibodies, all may contribute to the rejection of ABO-incompatible allografts. As described in the following section, the expansion of all these B-cell clones requires T-cell help, but these carbohydrate antigens cannot activate T cells. Such help may be provided by T-cell activation against major and minor HLA antigens. Such antigens may be highly immunogenic in some donor/recipient combinations and induce low T-cell activation, resulting in T-cell help to B cells producing antibodies to the incompatible carbohydrate antigens, despite the immunosuppression.

## Tolerance and accommodation to α-gal epitope as incompatible carbohydrate antigen in mice

### GT-KO mice as a model for anti-Gal immune response analysis

Analysis of A or B antibody production following successful transplantation of ABO-incompatible kidney or heart allografts demonstrated a lack of such production in some patients (i.e., immune tolerance) or production of such antibodies that do not induce graft rejection (Ishida et al., 2000; West et al., 2001; Urschel et al., 2013). These antibodies may be considered as inducing accommodation (Chopek et al., 1987; Garcia de Mattos Barbosa et al., 2018; Platt and Cascalho, 2023). Studies aimed at understanding the principles underlying the different types of immune response to incompatible carbohydrate antigens were performed in a mouse model grafted with such antigens. Since the α-gal epitope is the core of A and B antigens and, as such, it can be an immunogenic carbohydrate antigen in humans, it was of interest to determine whether this antigen can simulate an incompatible carbohydrate antigen in mice. Because the α-gal epitope is naturally synthesized in non-primate mammals, including mice, all these species are immunotolerant to it and cannot produce anti-Gal. However, α1,3-GT knockout (GT-KO) mice generated by Thall et al. (1995) and Tearle et al. (1996) lack α-gal epitopes. These mice produce anti-Gal following immunization with xenogeneic cell or cell membranes presenting α-gal epitopes, such as rabbit red cells (LaTemple and Galili, 1998), pig kidney membranes homogenates (Tanemura et al., 2000a), porcine cells (Cretin et al., 2002), or bacteria (Posekany et al., 2002) and protozoa presenting α-gal-like epitopes (Pearse et al., 1998). Without such immunization, GT-KO mice may only produce minimal amounts of anti-Gal IgM or no anti-Gal (Chong et al., 2000; Xu et al., 2002) because the mice are usually kept under sterile conditions and are fed sterile food, thus they cannot develop a bacterial flora that stimulates natural anti-Gal production. In contrast to GT-KO mice, GT-KO pigs not kept in sterile conditions readily produce the natural anti-Gal antibody already at the age of 6 weeks (Dor et al., 2004; Fang et al., 2012; Galili, 2013b). GT-KO mice producing anti-Gal served as recipients of grafts from syngeneic or semi-allogeneic WT mice that present the α-gal epitope as the incompatible carbohydrate antigen.

### The α-gal epitope cannot activate T cells, but anti-Gal production requires T-cell help

Carbohydrate antigens, which are oligosaccharides with a size of 3 units or more (e.g., ABO antigens and the α-gal epitope), are capable of binding to B cells with the corresponding B-cell receptor (BCR) but not to T-cell receptors (Ishioka et al., 1992; Speir et al., 1999; Avci et al., 2013). The binding of polysaccharides to B cells may result only in an immune response via the release of IgM without an isotype switch (Jackson et al., 2007). However, activation of B cells for proliferation, isotype switch, and differentiation into plasma cells and memory B cells, following the BCR binding carbohydrate antigen, all require help provided by activated helper T (Th) cells. Thus, coupling bacterial polysaccharides to an immunogenic protein can generate vaccines that achieve such Th-cell activation, which, in turn, enables full activation of B cells for the production of IgG and IgA antibodies and generation of memory B cells (Stein, 1992; Pletz et al., 2008). In view of these considerations, it was of interest to determine whether the α-gal epitope can bind to T cells with the corresponding TCR. GT-KO mice were immunized with pig kidney membranes (PKM) homogenate, which presents a high concentration of α-gal epitopes (Tanemura et al., 2000b). The immunized mice produced anti-Gal IgG in high titers as the immunogenic porcine peptides activated many Th cells, which provided help to the many anti-Gal B cells that engaged via their BCR the α-gal epitopes on the PKM (40). However, when immunization was performed with syngeneic WT mouse kidney membrane homogenate, which also presented α-gal epitopes, no anti-Gal IgG production was detected, and only marginal anti-Gal IgM production was detected (Tanemura et al., 2000a). Immunization with α-gal glycolipids purified from rabbit RBC membranes demonstrated weak anti-Gal IgM and no anti-Gal IgG production. These findings correlated with the demonstration of significant expansion of anti-Gal B cells (i.e., B cells that bound synthetic α-gal epitopes linked to BSA) only in mice immunized with porcine PKM (Tanemura et al., 2000a). These observations suggested that the α-gal epitope by itself cannot activate Th cells. This conclusion was further supported by the finding that co-incubation of lymphocytes that include memory T cells (i.e., spleen cells from GT-KO mice activated by porcine peptides following repeated immunizations with PKM) did not proliferate when co-incubated with syngeneic cells presenting α-gal epitopes (Tanemura et al., 2000a). However, these primed T cells proliferated when incubated with porcine cells presenting α-gal epitopes.

The requirement for T-cell help for the activation of anti-Gal B cells was further demonstrated by immunization of GT-KO mice with PKM concomitantly with the injection of anti-CD40L (a monoclonal antibody, which prevents T–B-cell interaction). Such immunization resulted in no production of anti-Gal IgG. However, anti-Gal IgM production was unaffected (Tanemura et al., 2000a). These findings implied that similar to immunization with α-gal glycolipids (glycolipids that do not activate T cells), inhibition of Th-cell activity prevents the production of anti-Gal IgG but enables some anti-Gal IgM production. The required T-cell help could also be provided by immunogenic proteins that are not associated with the PKM homogenates, for example, keyhole limpet cyanin (KLH), which provides multiple immunogenic peptides (Tanemura et al., 2000a).

#### Possible similarities in humans

In recipients of ABO mismatched allografts, T-cell immunosuppression by standard protocols may be expected to prevent Th help to B cells capable of producing pure anti-A, anti-B, anti-Gal/B, and anti-Gal/AB antibodies. The extent of T-cell inactivation under immunosuppression protocols may vary. In cases of highly immunogenic major and minor HLA molecules in various donor/recipient combinations, Th-cell activation may occur. An extreme example is that of diabetic patients transplanted with the kidney allograft and with porcine fetal islet cells, as described previously (Galili et al., 1995). In a few donor/recipient combinations, the immunogenicity of allograft HLA may result in low T-cell activation, despite immune suppression. This activation is not potent enough to cause T-cell-mediated rejection of the allograft. However, it may be sufficient for providing T-cell help that enables the activation of B-cell-producing antibodies against the incompatible carbohydrate antigen. It is possible that such weak activation suffices for enabling antibody-mediated gradual rejection of the graft by complement-dependent cytolysis (CDC) or antibody-dependent cell cytolysis (ADCC).

### Tolerance induction by heart grafts presenting α-gal epitopes in the absence of T-cell help

**FIGURE 4:**
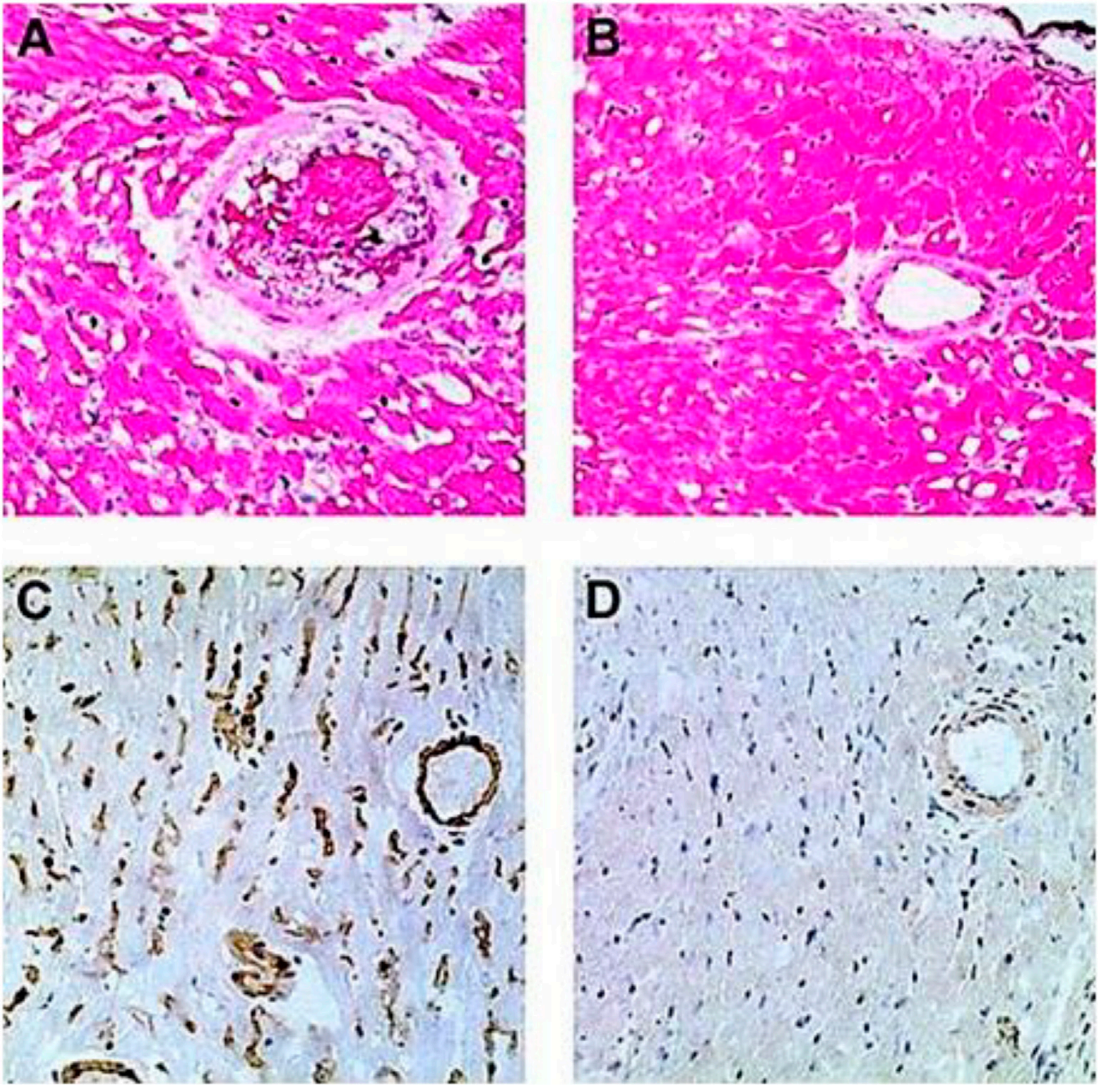
Induction of tolerance to the α-gal epitope on syngeneic WT mouse lymphocytes, as indicated by no rejection of heterotopically grafted mouse WT heart. **(A,C)** Hyperacute rejection within 30–60 min of WT heart grafted in GT-KO mice, which received 4 weekly PKM immunizations prior to the heart grafting. **(A)** The hearts were rejected as indicated by the occlusion of blood vessels and edema in peri-vascular regions. **(C)** The immunostained tissue displayed anti-Gal IgM binding to the endothelial cells of the grafted WT heart. Similar results were obtained with anti-IgG staining. **(B,D)** Hearts transplanted into mice tolerized by WT lymphocytes presenting α-gal epitopes that were administered 4 weeks prior to transplantation. The hearts were harvested 2 months after transplantation and were functioning despite three additional weekly PKM immunizations starting 1 week after grafting. **(B)** Normal myocardial structure. **(D)** No binding of IgM indicated by immunostaining with an anti-mouse IgM antibody. Similarly, no IgG binding was observed. **(A,B)** Hematoxylin–eosin staining (H&E); **(C,D)** immunostained with peroxidase coupled anti-mouse IgM antibodies (×200). From Ogawa et al. (2003), with permission.

GT-KO mice were transplanted heterotopically in the abdomen with syngeneic WT mouse hearts (i.e., heart grafts presenting multiple α-gal epitopes as an incompatible carbohydrate antigen) (Ogawa et al., 2003; Ogawa et al., 2004a). These mice served as a model for determining whether repeated encounters of anti-Gal B cells with α-gal epitopes on the graft (i.e., engaging the BCRs with α-gal epitopes) in the absence of T-cell help affect these B cells. Previous studies have demonstrated the long-term survival of such hearts in naïve GT-KO mice. However, if the mice were immunized prior to grafting by PKM or by *Leishmania* to produce anti-Gal, the hearts were rejected by anti-Gal-mediated CDC and ADCC in a process called “hyperacute rejection” of the graft within 30 min to several hours (Pearse et al., 1998; Ogawa et al., 2003). This rejection is similar to the hyperacute rejection of an ABO-incompatible heart or kidney if the corresponding antibodies are not removed prior to grafting by plasmapheresis or by adsorption of the antibodies on columns presenting the incompatible carbohydrate antigen. However, if repeated weekly PKM immunizations of grafted mice started 4 weeks following transplantation, no rejection of the transplanted heart was observed, and no production of elicited anti-Gal was detected (Figures 4B, D) (Ogawa et al., 2004a). This finding suggested that in the absence of T-cell help, the repeated encounters of the BCR of naïve anti-Gal B cells with α-gal epitopes on the grafted WT heart endothelial cells tolerize these B cells.

The observed tolerance induction on naïve anti-Gal B cells raised the question of whether a similar tolerance can also be induced on memory anti-Gal B cells. Formation of memory anti-Gal B cells is feasible by three–five immunizations of the mice with PKM. However, grafting of the immunized mice with a WT mouse heart results in hyperacute rejection of the graft (Figures 4A, C). Although the removal of the natural anti-blood-group antibodies is feasible in humans, technically, it is not possible in mice. Thus, to have GT-KO mice transplanted with WT hearts and memory anti-Gal B cells, unimmunized GT-KO mice were heterotopically grafted with WT mouse hearts. Two weeks later, the mice were irradiated for the destruction of the self-hematopoietic and lymphoid systems. Subsequently, the mice received by adoptive-transfer 20 × 10^6^ splenocytes from PKM immunized GT-KO mice (i.e., adoptive transfer of lymphocytes that included memory anti-Gal B cells from PKM-primed mice). The mice also received 20 × 10^6^ bone marrow cells from unimmunized GT-KO mice for regenerating the hematopoietic system. PKM immunizations on a weekly basis were delivered to the mice, starting at various days after the adoptive transfer of the memory anti-Gal B cells. Anti-Gal production was determined by ELISA with α-gal BSA as a solid-phase antigen.

#### WT heart rejection after PKM immunization 24 h after adoptive transfer of memory anti-Gal B cells

PKM immunization of the mice 24 h after the adoptive transfer resulted in the activation of Th cells (by porcine immunogenic peptides) and memory anti-Gal B cells (by porcine α-gal epitopes) and the production of elicited anti-Gal IgM, IgG1, and IgG3, which mediated the rejection of the WT hearts 3–7 days after immunization (Ogawa et al., 2004a). As shown in Figure 4, this rejection was mediated by antibodies binding to the endothelial cells and the peri-vascular cardiomyocytes of the WT heart. Moreover, in the presence of complement, these anti-Gal antibodies effectively induced *in vitro* CDC of mouse cells presenting α-gal epitopes even in a serum dilution of 1:1,000.

#### Tolerance induction after PKM immunization 4 weeks after adoptive transfer

PKM immunization of the grafted mice 4 weeks after adoptive transfer did not cause rejection of the WT heart grafts. The heart function was not impaired even after three additional weekly PKM immunizations (Ogawa et al., 2004a). When the functioning transplanted WT hearts were explanted after 100 days and immunostained, they displayed patent blood vessels with no immunoglobulins bound to them, as shown in Figures 4B, D. These findings suggest that both memory and naïve anti-Gal B cells were tolerized as a result of repeated encounters of their BCRs with α-gal epitopes on the endothelial cells of the WT heart grafts for a prolonged period of 4 weeks and in the absence of T-cell help. This tolerance was not the result of anergy of anti-Gal B cells that are unresponsive to antigen stimulation and may reactivate in the absence of the tolerizing antigen (Yarkoni et al., 2010; Burnett et al., 2019). This was demonstrated by a second adoptive transfer of lymphocytes from the tolerized mice to naïve recipients. These recipients were immunized twice with PKM, starting 2 weeks after the second adoptive transfer, but failed to produce anti-Gal (Ogawa et al., 2004a). This result implies that no anergic anti-Gal B cells could recover from the state of anergy during the 2 weeks in the secondary recipient in the absence of α-gal epitopes. Thus, the observed tolerance to α-gal epitopes on the WT mouse vascular wall was likely to be the result of the elimination of naïve and memory anti-Gal B cells by either apoptosis following multiple engagements of their BCR with α-gal epitopes on the WT endothelial cells or Ig receptor editing that alters the specificity of their BCR (Radic and Zouali, 1996). The observed permanent state of tolerance (>100 days, despite repeated PKM immunizations) strongly suggests that new anti-Gal B cells emerging in the bone marrow “regard” the α-gal epitopes on the graft as a self-antigen and thus are tolerized by it.

#### Accommodation induction after PKM immunization 1–2 weeks after adoptive transfer

The effects of intermediate time of memory anti-Gal B-cell exposure to α-gal epitopes on the WT heart graft for 1 or 2 weeks instead of 4 weeks were also tested. No rejection of hearts was observed following PKM immunizations that started for 1 or 2 weeks. However, the transplanted mice produced anti-Gal antibodies, which readily bound to the endothelial cells of the graft, without causing any damage to the blood vessels or the myocardium of the graft (Mohiuddin et al., 2003a). Such production of antibodies against the incompatible carbohydrate antigen of the graft without damaging the graft structure or function for months has been referred to as “immune accommodation” (Alexandre et al., 1987; Bannett et al., 1987; Chopek et al., 1987; Latinne et al., 1989; Park et al., 2003; Garcia de Mattos Barbosa et al., 2018; Platt and Cascalho, 2023). Immunohistological comparison of the grafts rejected on Day 7 in mice receiving PKM immunization 24 h after adoptive transfer and grafts of accommodated hearts from mice immunized with PKM on Day 7 and explanted on Day 21 revealed the following differences: the rejected hearts displayed binding of IgM, IgG1, and IgG3 to blood vessels, whereas the accommodated hearts displayed binding of IgM, IgG1, and IgG2b but not IgG3. ELISA analysis of anti-Gal IgG subclasses demonstrated a much higher activity of anti-Gal IgG2b in the accommodating mice than in the rejecting mice or in mice that just received four PKM immunizations and no graft or adoptive transfer of lymphocytes (Mohiuddin et al., 2003a). As indicated previously, high *in vitro* CDC activity against α-gal presenting cells was observed in the sera of the mice immunized by PKM 24 h after adoptive transfer, whereas no cytolytic activity was detected in the sera of accommodating mice (Mohiuddin et al., 2003a). These accommodation studies suggested that a large proportion of anti-Gal B cells repeatedly encountering α-gal epitopes in grafted WT hearts for 7 days in the absence of T-cell help undergo isotype switch for the production of accommodating anti-Gal IgG2b antibody. This antibody binds to the α-gal epitopes and prevents complement activation and graft rejection by cytolytic anti-Gal antibodies. Repetition of these experiments in mice receiving the first of the two PKM immunizations 14 days after adoptive transfer resulted in accommodation induction in only 60% of the mice, whereas the remaining 40% displayed immune tolerance, similar to that described previously for mice immunized with PKM 4 weeks after adoptive transfer (Mohiuddin et al., 2003a; Ogawa et al., 2004a). Notably, some of the mice with the accommodated heart were transplanted in the cervical area with a second WT heart by connecting the WT aorta with the GT-KO carotid artery and WT pulmonary artery with the GT-KO internal jugular vein. These mice also received a third PKM immunization 1 week before the transplantation of the second heart, and high titers of anti-Gal were confirmed at the time of transplantation. The second heart was not rejected and functioned for more than 2 additional months despite the high titers of anti-Gal (Mohiuddin et al., 2003a). As the second heart graft was not exposed to the accommodating process, these observations strongly suggest that the accommodation of the first transplanted WT hearts was not because of decreased expression of α-gal epitopes during the accommodation period.

#### Possible similarities in humans

The spectrum of immune responses to the incompatible α-gal epitopes described previously in GT-KO mice from hyperacute rejection via accommodation to immune tolerance seems to exist also in humans. Early attempts at transplantation of ABO-incompatible kidney allografts indicated that many of these allografts were subjected to hyperacute rejection by anti-blood-group A or B antibodies binding to the incompatible B or A antigen, respectively, on the endothelial cells of the graft (Starzl et al., 1964). This binding results in complement activation, cytolysis, the rapid collapse of the vascular bed, and hyperacute rejection. With the development of immunosuppressive drugs preventing T-cell activation, plasmapheresis, methods for removal of anti-A or anti-B antibodies, and a decrease in the activity of the immune system by splenectomy, it was shown in the 1980s that the rejection of ABO-incompatible kidney allograft was prevented in many of the patients, although they produced anti-A or anti-B antibodies. These were accommodating antibodies bound to the endothelial cells of the graft vascular system but did not mediate complement activation and cytolysis of the graft cells (Alexandre et al., 1987; Bannett et al., 1987; Chopek et al., 1987; Latinne et al., 1989; Park et al., 2003; Garcia de Mattos Barbosa et al., 2018; Platt and Cascalho, 2023). In more recent studies, pre-transplantation-specific removal of anti-A or anti-B antibodies in some patients was performed by adsorption of the plasma in columns containing beads presenting the corresponding A or B antigen (Tanabe et al., 1998; Tydén et al., 2003; Genberg et al., 2007). Rituximab (anti-CD20 antibody eliminating B cells) is used in some centers instead of splenectomy (Sonnenday et al., 2004). Clinical studies reported the production of non-rejecting (i.e., accommodating) antibodies or the lack of antibody production against the incompatible A or B antigen (i.e., immune tolerance) in ABO mismatched kidney (Alexandre et al., 1987; Bannett et al., 1987; Chopek et al., 1987; Latinne et al., 1989; Ishida et al., 2000; Park et al., 2003; Garcia de Mattos Barbosa et al., 2018; Platt and Cascalho, 2023) or heart allograft recipients (West et al., 2001; Urschel and West, 2016; Issitt et al., 2021). It is suggested that the observed accommodation and tolerance are associated with the length of time for repeated encounters of the BCRs on B cells capable of producing anti-A or anti-B antibodies with the corresponding incompatible A or B antigen in the absence of T-cell help (due to the immunosuppression treatment). In an analogy with the accommodation and tolerance to incompatible α-gal epitopes in GT-KO mice, this repeated encounter may result in some patients in production of accommodating anti-A or anti-B antibodies. However, it will lead to complete tolerance to the incompatible A or B antigen in other patients. The ultimate result of rejecting antibody production, accommodation, or tolerance may depend on several variable factors, including the success of pre-transplantation elimination of the circulating natural antibodies, the extent of T-cell suppression in the individual patient, the amount of effective multiple encounters of B cells with the incompatible antigen in the allograft, and T-cell immunogenicity of the allograft in the particular donor/recipient combination.

## Induction of tolerance by lymphocytes and bone marrow cells presenting α-gal epitopes

### Tolerance induction on naïve anti-Gal cells

The observations on tolerance and accommodation induction by α-gal epitopes on WT heart in the absence of T-cell help raise the question of whether similar effects on the immune response can be induced by α-gal epitopes on cells other than WT endothelial cells. Syngeneic lymphocytes were obtained from the spleens of WT mice. Naïve GT-KO mice received intravenously 2 × 10^6^ or 20 × 10^6^ syngeneic WT lymphocytes presenting α-gal epitopes from C57BL/6 mice syngeneic to GT-KO mice. The mice further received four weekly PKM immunizations, 14 days after the administration of these α-gal-presenting cells. The mice in both groups of recipients displayed subsequently complete absence of anti-Gal production. This implied that mice receiving WT lymphocytes were tolerized to the α-gal presented on these cells (Ogawa et al., 2003). This tolerance was specific to the α-gal epitope since the immunized mice displayed robust antibody production to proteins and peptides within the immunizing PKM. ELISPOT analysis of anti-Gal producing B cells demonstrated the absence of such cells in the tolerized and PKM immunized mice, suggesting the elimination of anti-Gal B cells following their repeated BCR engaging with α-gal epitopes on the WT lymphocytes. A similar tolerance induction associated with the elimination of anti-Gal B cells was reported in GT-KO mice transplanted with syngeneic WT bone marrow cells (Yang et al., 1998). Thus, tolerance induction on anti-Gal B cells seems feasible when the α-gal epitope is presented on a variety of cells in the absence of T-cell activation.

### Tolerance induction on memory anti-Gal B cells

**FIGURE 5:**
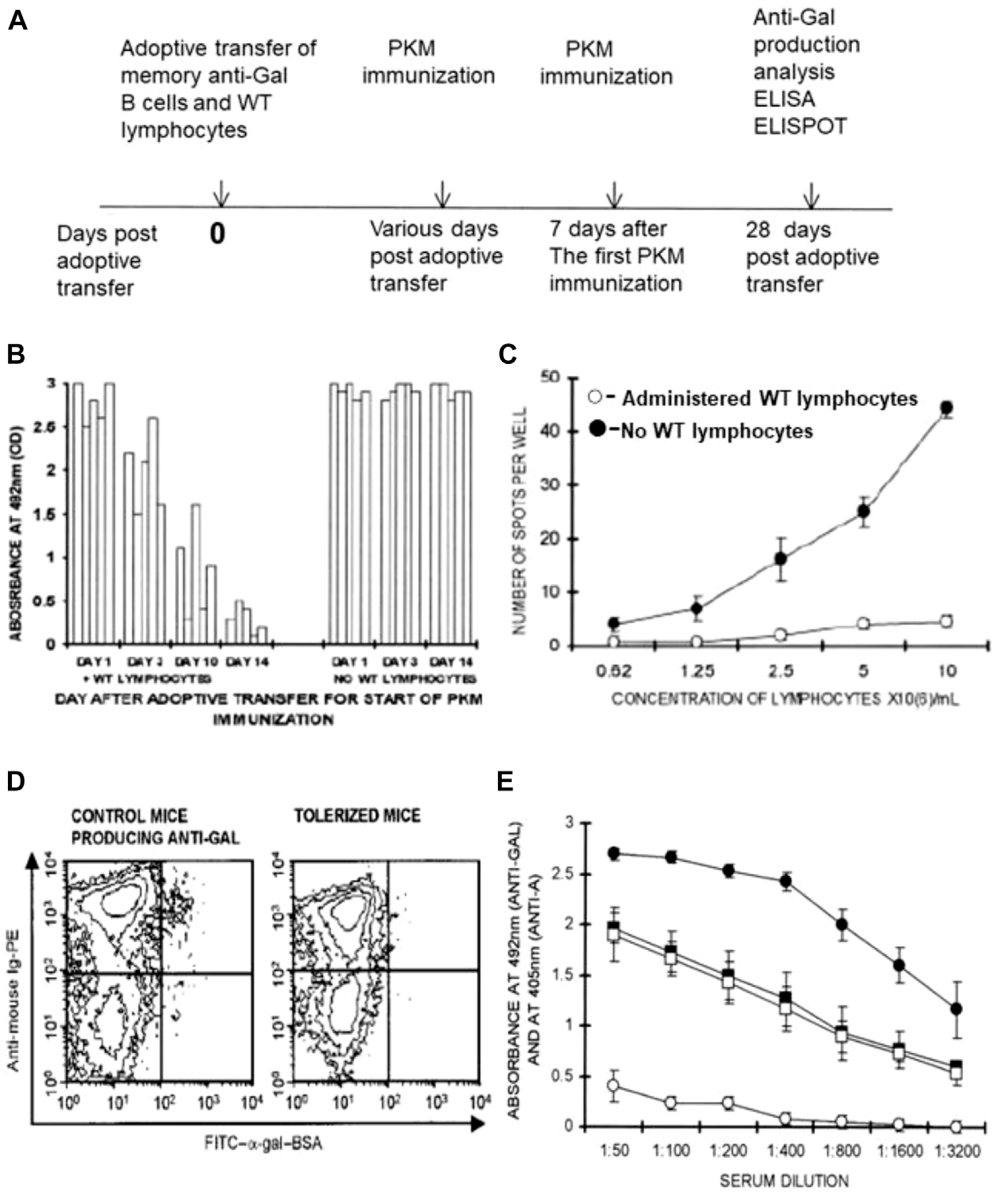
Induction of immune tolerance to α-gal epitopes in GT-KO mice by elimination of memory anti-Gal B cells following administration of WT lymphocytes presenting α-gal epitopes. **(A)** Timeline for the induction of tolerance on memory anti-Gal B cells. **(B)** Time required for tolerance induction. Irradiated GT-KO mice received 20 × 10^6^ lymphocytes, including memory anti-Gal B-cells, naïve GT-KO bone marrow cells, and WT lymphocytes or no WT lymphocytes (control group). The mice further received two PKM immunizations, the first of which was at 1, 3, 10, and 14 days. The second PKM immunization and ELISA and ELISPOT (both with α-gal BSA as a solid-phase antigen) were performed as in **(A)**. Absorbance values are presented at a serum dilution of 1:100. Each column represents one out of five mice in each group. **(C)** ELISPOT analysis of anti-Gal secretion in tolerized *versus* control mouse spleen cells was performed with α-gal BSA as a solid-phase antigen. Mice tolerized by WT lymphocytes (○), or control mice receiving no WT lymphocytes (●). Means ± SE (*n* = 5). **(D)** Flow cytometry identification of anti-Gal B cells among B cells by double staining with FITC-α-gal BSA (green) and PE-anti-mouse Ig (red-staining of all B cells). Control and tolerized mice, as in **(C)**. Note that as many as ∼1% of B cells bound α-gal epitopes of α-gal BSA in the control mice (i.e., anti-Gal B cells), whereas almost no such B cells were detected in the tolerized mice. **(E)** Tolerance induction on memory anti-Gal B cells does not affect B cells producing anti-blood-group A antibody. The study was performed as in **(A)**. However, both experimental and control mice received a mixture of memory anti-Gal B cells and memory anti-blood-group A B cells from mice immunized four times with blood-group A red cells. The first of the two PKM and blood-group A red cell immunizations was delivered on Day 14. Anti-blood-group A antibody production was assayed by ELISA with A red cell membranes as a solid-phase antigen. Anti-Gal antibody production was assayed by ELISA with α-gal BSA as a solid-phase antigen. (□, ○) Experimental mice also receiving WT lymphocytes. (■, ●) Control mice receiving no WT lymphocytes. (□, ■) Anti-blood-group A IgG production. (○, ●) Anti-Gal IgG production. Note that anti-Gal B cells were tolerized by the WT lymphocytes, whereas anti-blood-group A B-cell-produced anti-A antibodies were not affected. Means ± SE (*n* = 5). From Mohiuddin et al. (2003b), with permission.

The analysis of tolerance induction on memory anti-Gal B cells was performed as described previously in irradiated GT-KO mice that received by adoptive transfer a mixture of 20 × 10^6^ lymphocytes from PKM-primed GT-KO mice (i.e., lymphocytes including memory anti-Gal B cells) and 20 × 10^6^ lymphocytes from WT mice, as well as bone marrow cells from GT-KO mice. After 14 days, mice received a PKM immunization that was followed by a second PKM immunization on Day 21. Anti-Gal production was determined on Day 28 after adoptive transfer (Figure 5A) (Mohiuddin et al., 2003b). These treated mice displayed no production of anti-Gal, whereas PKM-immunized mice receiving memory anti-Gal B cells and no WT lymphocytes displayed a robust anti-Gal response (Figure 5B). ELISPOT studies indicated that in the anti-Gal-producing mice >40 anti-Gal-producing cells were detected among 10^6^ splenocytes (10^7^ cells/ml), whereas in the mice that did not produce the antibody, only <5 anti-Gal-producing cells were detected per 10^6^ splenocytes (Figure 5C).

The identification of memory anti-Gal B cells in mice in Figure 5C was performed by flow cytometry of B-cell binding of labeled α-gal-BSA to their BCRs (Figure 5D). As many as ∼1% of the B cells that displayed binding of α-gal BSA were found on Day 28 in control mice that received memory B cells from PKM-primed mice but no WT lymphocytes. In contrast, only a marginal background level of anti-Gal B cells was found in the spleens of mice that received both memory anti-Gal B cells and WT lymphocytes (Figure 5D). Data in Figures 5B–D strongly suggest that the memory anti-Gal B cells were eliminated or underwent Ig receptor editing in the mice tolerized by the α-gal epitopes presented on WT lymphocytes. The tolerized mice were further transplanted heterotopically with the syngeneic WT mouse heart and received additional PKM immunizations. No rejection of the transplanted hearts was observed since the grafted mice conserved the state of tolerance to the α-gal epitope (Mohiuddin et al., 2003b).

The observed tolerance induction was highly specific to memory anti-Gal B cells and did not affect antibody production by memory B cells specific to the blood-group A antigen. This could be demonstrated by performing adoptive transfer into irradiated mice of lymphocytes including memory anti-Gal B cells from PKM-primed mice and lymphocytes including memory anti-blood-group A B cells from mouse donors that received four immunizations by human blood-group A red cells. In addition, the irradiated recipients received WT lymphocytes and GT-KO bone marrow cells, as described previously. Control mice received a similar mixture of memory anti-Gal B cells, memory anti-A B cells, and GT-KO bone marrow cells but no tolerizing WT lymphocytes. PKM and blood-group A red cell immunizations were performed on Days 14 and 21 after adoptive transfers and antibody production was assayed by ELISA on Day 28, using α-gal BSA and A-red cell ghosts as solid-phase antigens. Sera were adsorbed on blood-group O red cells for the removal of anti-human red cell antibodies that were not anti-A. Although control mice (not receiving WT lymphocytes) produced anti-Gal and anti-A antibodies, the mice receiving WT lymphocytes produced anti-A antibodies but no anti-Gal antibodies (Figure 5E). These findings implied that only memory anti-Gal B cells were tolerized, whereas the activity of anti-A B cells was not affected.

In an attempt to determine how long it takes for the induction of tolerance by WT lymphocytes on memory anti-Gal B cells, the experiment illustrated in Figure 5A was repeated. However, the day of the first PKM immunization was the 1st, 3rd, and 10th days instead of the 14th day after the adoptive transfer, and the second PKM immunization was delivered 1 week after the first immunization in each group. A significant tolerizing effect of α-gal epitopes on the memory anti-Gal B cells was observed in two of the mice by Day 10 and displayed by Day 14 in all five mice. However, no significant prevention of anti-Gal production was observed in mice immunized with PKM on Days 1 and 3 (Figure 5B). This implied that the activation of multiple T cells by the immunogenic porcine peptides of the PKM resulted in “rescuing” memory anti-Gal B cells from elimination even after 3 days of encounters between the α-gal epitopes on WT lymphocytes and BCRs on memory anti-Gal B cells. However, 10 days of such encounters resulted in tolerance induction on these B cells in some of the mice, and 14 days sufficed for tolerance induction in all the treated mice (Mohiuddin M. et al., 2003).

The rescue of memory anti-Gal B cells by immunization with PKM on Days 1 and 3 in Figure 5B was the result of the activation of Th cells by multiple porcine immunogenic peptides (Tanemura et al., 2000a). Thus, it was of interest to determine whether H-2 antigens can also induce T-cell activation that may rescue memory anti-Gal B cells from being tolerized by α-gal epitopes. This was studied by the use of semi-allogeneic H-2bxd WT lymphocytes obtained from F1 C57BL/6 × BALB/c (H2-bxd) offspring. WT lymphocytes from these mice were introduced into mice that received memory anti-Gal B cells and immunized with PKM on Days 14 and 21 (as in Figure 5A). The H-2d alloantigen on the F1 WT lymphocytes sufficiently activated T cells to provide the help required for rescuing memory anti-Gal B cells from being tolerized by α-gal epitopes on the WT lymphocytes (Mohiuddin et al., 2003b).

#### Possible similarities in humans

As discussed previously, the effect of Th-cell activation by an H-2 alloantigen may be of significance in human donor/recipient combinations, in which, in addition to the expression of an incompatible A or B antigen, the grafts present major or minor HLA antigens, which may induce weak activation of the recipient’s T cells despite immunosuppression. Such a low T-cell activation may suffice for enabling the activation of the recipient’s B cells capable of producing anti-A or anti-B antibodies against an incompatible carbohydrate antigen.

### Tolerance induction by autologous lymphocytes engineered to present α-gal epitopes

The effective tolerance induction by WT syngeneic lymphocytes presenting α-gal epitopes raised the possibility that autologous GT-KO mouse lymphocytes engineered to present α-gal epitopes may have a similar tolerizing effect to the syngeneic WT lymphocytes. If successful, such a study suggests that autologous human lymphocytes engineered to preset blood-group A or B antigen can induce tolerance to these antigens prior to transplantation of an allograft from a live donor (e.g., a kidney graft from a relative donor). Synthesis and presentation of α-gal epitopes on GT-KO mouse lymphocytes was achieved by *in vitro* transduction of these cells for 4 h with a replication-defective adenovirus vector containing the mouse α1,3-GT gene *GGTA1*, referred to as AdαGT (Deriy et al., 2002). The presentation of α-gal on the transduced cells within 24 h after transduction is similar to that on WT mouse lymphocytes (Ogawa et al., 2004b).

A total of 20 million GT-KO mouse lymphocytes transduced with AdαGT or control lymphocytes transduced with the “empty” adenovirus vector were administered intravenously to naïve GT-KO mice. Administration of the transduced lymphocytes was repeated on Days 4 and 9 to overcome the possibility that the α1,3-GT gene *GGTA1* in AdαGT transduced cells may be destroyed with time by nucleases. The repeated administration of the transduced lymphocytes provides autologous circulating lymphocytes presenting α-gal epitopes for at least 13 days. Starting on Day 14, the mice received four weekly PKM immunizations, and anti-Gal production was analyzed 1 week after the last immunization. No anti-Gal production was detected in mice receiving AdαGT transduced lymphocytes, whereas mice receiving control lymphocytes displayed a robust anti-Gal production (Ogawa et al., 2004b). Similar tolerance induction was observed in GT-KO mice that received autologous bone marrow cells transduced *in vitro* with the *GGTA1* gene (Bracy et al., 1998; Bracy and Iacomini, 2000).

In order to determine whether GT-KO mouse lymphocytes transduced with AdαGT can tolerize memory anti-Gal B-cells, the study was repeated, as shown in Figure 5A. However, the irradiated mice received 20 × 10^6^ transduced GT-KO lymphocytes instead of syngeneic WT lymphocytes. As mentioned previously, the administration of the transduced lymphocytes was repeated on Days 4 and 9. Following two PKM immunizations on Days 14 and 21, the mice were assayed on Day 28 for anti-Gal production. No anti-Gal production was detected in mice receiving lymphocytes transduced with AdαGT, whereas mice receiving the control lymphocytes transduced with the empty adenovirus vector displayed extensive anti-Gal production that was readily detected by ELISA even at a serum dilution of ∼1:1,000 (Ogawa et al., 2004b). The mice receiving AdαGT transduced lymphocytes were further transplanted heterotopically on Day 28 with a WT heart. Sixty-five percent of the transplanted hearts continued to function for 45–100 days until the mice were euthanized for histological inspection of the hearts. This activity of the grafted WT hearts continued despite four additional weekly PKM immunizations. The remaining 35% of the mice died after 62–64 days for unknown reasons. Among mice receiving lymphocytes transduced with empty adenovirus vector, transplanted WT hearts were rejected by the produced anti-Gal antibody within 0.5–18 h. These studies with AdαGT-transduced autologous lymphocytes indicated that the tolerizing efficacy of autologous GT-KO mouse lymphocytes engineered to present α-gal epitopes is similar to that of WT mouse lymphocytes.

#### Possible significance in humans

The ability of autologous lymphocytes transduced with AdαGT to present incompatible carbohydrate antigens and the tolerance induction by such lymphocytes, as described previously, may be considered a potential tool for tolerizing the immune system of recipients receiving ABO-incompatible grafts for inducing tolerance to the incompatible blood-group A or B antigen. The significance of such a tolerizing system and a theoretical example for this treatment are detailed in the following section.

## Suggested method for induction of tolerance to ABO-incompatible antigens in allograft recipients

The protocols that are presently used for the transplantation of ABO-incompatible grafts include a stage of decreasing the number of lymphocytes in the recipient prior to transplantation, by splenectomy (Alexandre et al., 1987; Bannett et al., 1987; Chopek et al., 1987; Latinne et al., 1989; Park et al., 2003; Garcia de Mattos Barbosa et al., 2018; Platt and Cascalho, 2023) or by administration of rituximab, which mediates non-specific destruction of B cells (Sonnenday et al., 2004). The risk associated with these methods is the decrease in antibody production against opportunistic infections following the transplantation procedure. In view of the success of autologous lymphocytes engineered to present α-gal epitopes in inducing tolerance to this antigen (Ogawa et al., 2004b), a similar method may be able to induce tolerance to incompatible blood-group A or B antigen in allograft recipients. A hypothetical example of such tolerance induction is blood-group A or O individuals who will receive a blood-group B kidney allograft. The suggested treatment may include the following steps: 1) the treatment is initiated with standard immune suppression of T cells to minimize activation of T cells against various antigens during the tolerance induction procedure. 2) Two weeks prior to transplantation, the anti-blood-group B antibody in the patient’s blood is removed by passing the plasma through a column of beads (e.g., silica beads) that presents synthetic blood-group B antigen. This adsorption of the plasma may be performed at low temperatures to minimize complement activation. Alternatively, anti-blood-group B antibodies are removed by plasmaphereses. 3) Mononuclear cells are isolated from the patient’s blood, concomitant with the removal of the anti-blood-group B antibody. 4) Mononuclear cells are transduced *in vitro* with a replication-defective adenovirus containing the blood-group B transferase gene (AdBT) (Yamamoto et al., 1990). 5) The transduced cells are re-administered into circulation. 6) Steps 2–4 are repeated on Days 4 and 9. If the treated patient is found to produce an anti-blood-group B antibody, the removal of the antibody is repeated prior to the re-administration of the transduced lymphocytes. 7) If no production of anti-B antibody is detected within 2 weeks after treatment, the kidney allograft presenting blood-group B antigen is transplanted under a standard allograft immunosuppression protocol. A similar procedure is performed with AdAT in a blood-group B or O patient receiving a kidney presenting blood-group A antigen and with both AdAT and AdBT in a blood-group O patient receiving a blood-group AB allograft. It is hypothesized that in the absence of T-cell help due to immune suppression, the corresponding B cells with BCR of the incompatible carbohydrate antigen will be eliminated, resulting in specific immune tolerance to that antigen. The immune system may continue to “regard” the incompatible A or B antigen as a tolerizing self-antigen as long as it is presented by the allograft.

## Potential harnessing of the anti-Gal/α-gal epitope interaction for therapies in various clinical settings

The aforementioned sections describe studies of the anti-Gal/α-gal epitope interaction, which provide a method for overcoming the detrimental effects of the incompatible A or B antigen in allograft recipients. However, as anti-Gal is abundantly produced in large amounts in all humans (Galili et al., 1984), the anti-Gal/α-gal epitope interaction may be harnessed for a number of therapies, called “α-gal therapies,” which were demonstrated to be successful in GT-KO mice and GT-KO pigs. This section summarizes some of the suggested therapies and provides references for readers who may be interested in obtaining additional information on such therapies.

**FIGURE 6:**
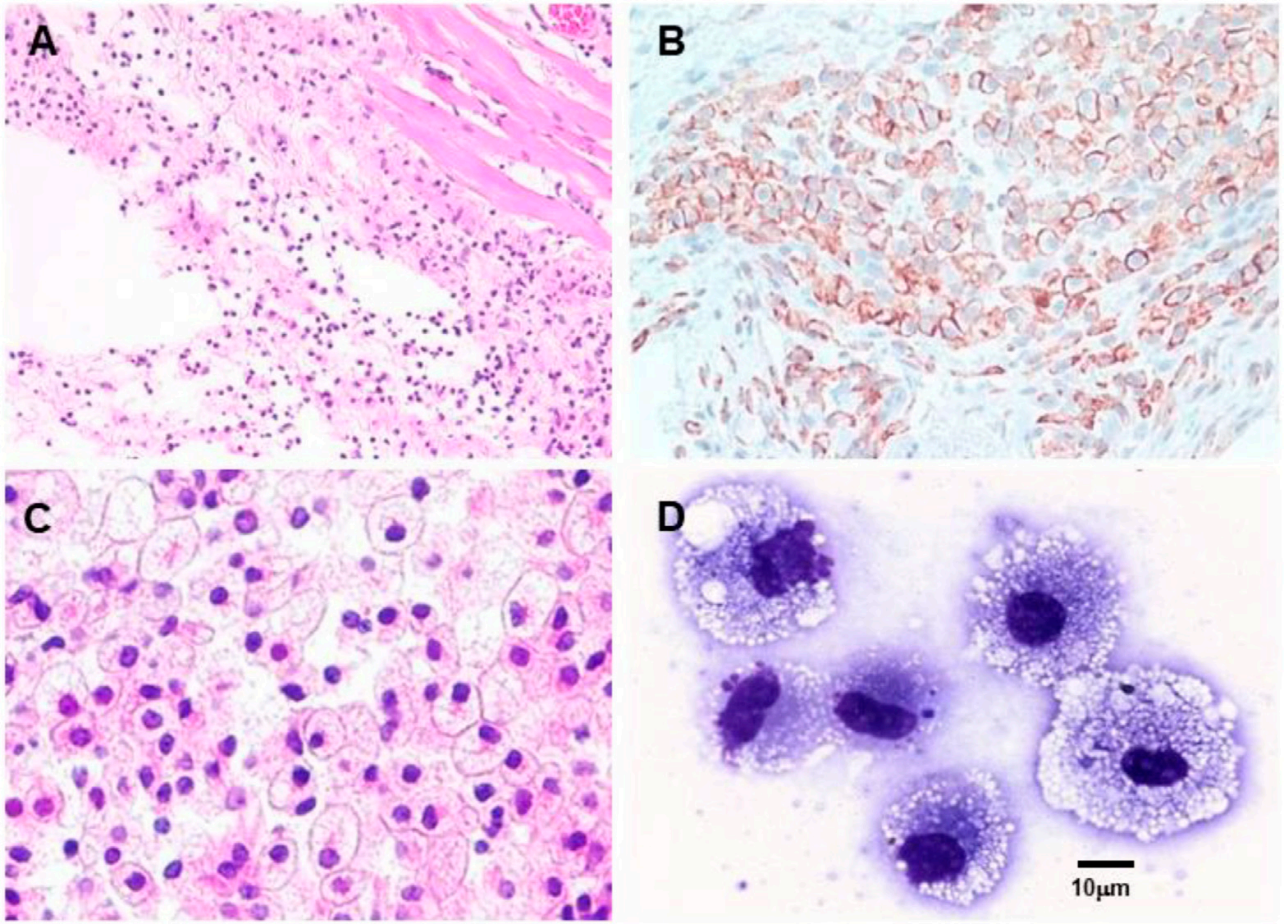
Intradermal recruitment of macrophages in anti-Gal-producing GT-KO mice by 10 mg α-gal nanoparticles. **(A)** Macrophage recruitment 24 h after injection of α-gal nanoparticles. The injection site is the empty area in which nanoparticles were eliminated during the fixation process (H&E ×100). **(B)** Identification of the recruited cells as macrophages by immunostaining on Day 4 after injection with the macrophage-specific peroxidase coupled-anti-F4/80 antibody (×200). **(C)** Macrophages at the injection site on Day 7. Macrophages are large with ample cytoplasm (H&E ×400). **(D)** Macrophages recruited into a polyvinyl alcohol sponge disc containing 10 mg α-gal nanoparticles 7 days after subcutaneous implantation into a GT-KO mouse. (Wright staining, ×1,000). Reproduced from Galili U. book “The natural anti-Gal antibody as foe turned friend in medicine,” Academic Press/Elsevier, London, 2018, with permission.

The two basic characteristics of anti-Gal potentially of use in various therapies are as follows: 1) anti-Gal/α-gal epitope interaction effectively activates the complement system. In addition to inducing complement-mediated cell cytolysis (as observed in the hyperacute rejection of porcine xenografts in primates producing anti-Gal), complement activation results in the formation of C5a and C3a complement cleavage peptides, which are among the most potent physiologic chemotactic factors directing the recruitment of antigen-presenting cells (APCs) such as macrophages and dendritic cells to the site of anti-Gal/α-gal epitope interaction. This is illustrated in Figure 6, which describes the results of the intradermal administration of biodegradable nanoparticles presenting α-gal epitopes in anti-Gal-producing GT-KO mice. Within 24 h, a clear migration of macrophages was observed at the injection site (Figure 6A). The number of macrophages increases by Day 4 (Figure 6B) and peaks by Day 7 (Figure 6C). 2) Anti-Gal binding to particulate materials or glycoproteins presenting α-gal epitopes is followed by interaction between the Fc “tail” of the immunocomplexed anti-Gal and Fc receptors on macrophages and dendritic cells, followed by the effective uptake of such immune complexes by these cells. This interaction further activates the macrophages, resulting in their increased size, as shown in Figure 6D, following the uptake of multiple anti-Gal-coated α-gal nanoparticles. The macrophages also reside at the injection site after 2 weeks but completely disappear after 3 weeks without altering the skin structure (Wigglesworth et al., 2011).

**FIGURE 7:**
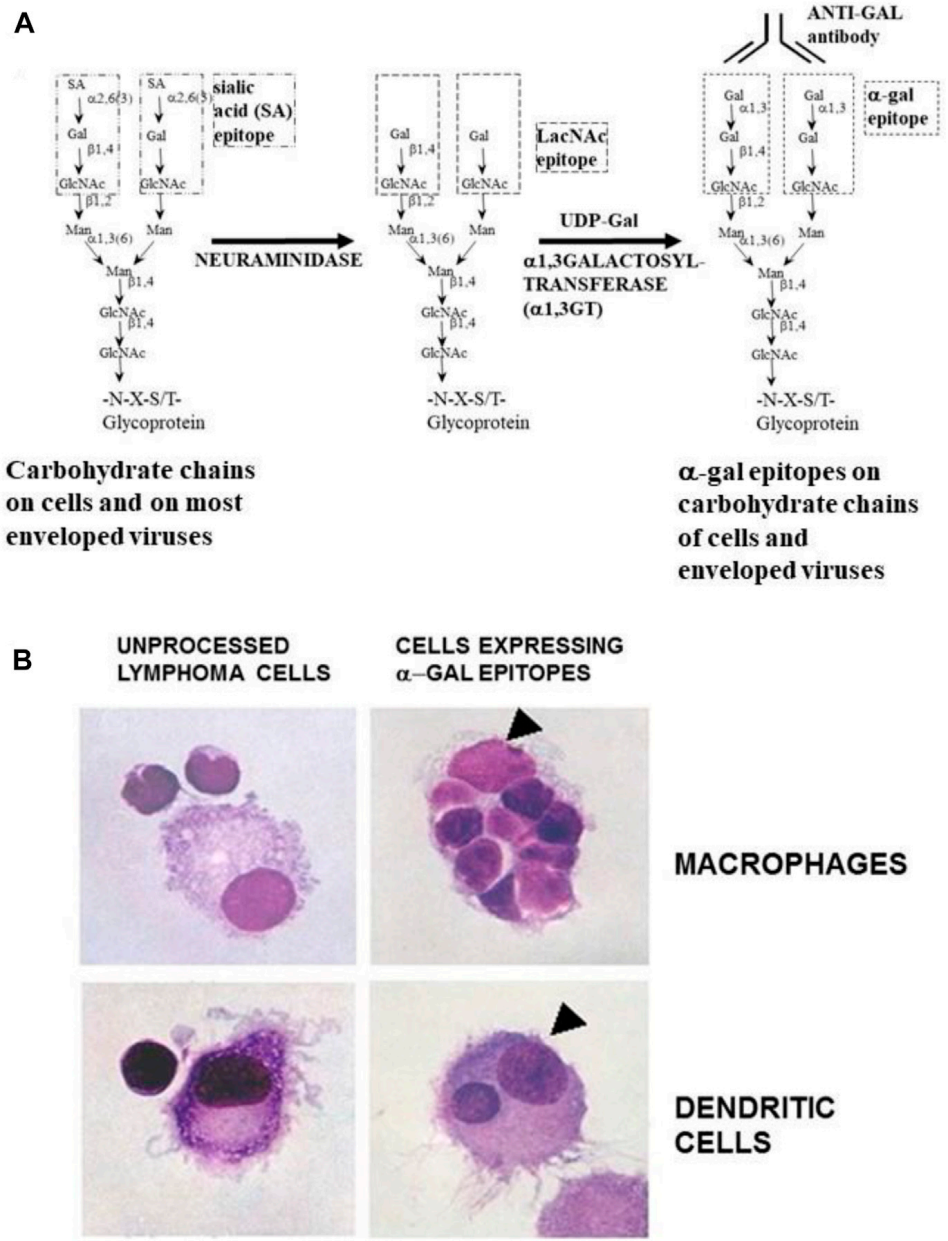
Anti-Gal-mediated targeting of α-gal presenting human lymphoma cells to APC. **(A)** Synthesis of α-gal epitopes on human tumor cells studied. (Left chain) A representative N-linked carbohydrate chain capped by sialic acid (SA). (Center chain). Sialic acid is removed by neuraminidase, thereby exposing the penultimate Galβ1-4GlcNAc-R called N-acetyllactosamine (LacNAc) (Center chain). The recombinant glycosylation enzyme α1,3-galactosyltransferase (rα1,3-GT) links galactose provided by sugar donor uridine diphosphate galactose (UDP-Gal) to the carbohydrate chain, resulting in the synthesis of α-gal epitopes (Galα1-3Galβ1-4GlcNAc-R), which readily bind the anti-Gal antibody (Left chain). A similar glycoengineering for the expression of α-gal epitopes can be performed in enveloped viruses. **(B)** *In vitro* demonstration of anti-Gal-mediated uptake of human lymphoma cells by autologous APC. Freshly obtained lymphoma cells were subjected to α-gal epitope synthesis, as described in **(A)**. The lymphoma cells with or without α-gal epitopes were incubated with autologous anti-Gal for 30 min then for 2 h at 37°C with autologous macrophages or dendritic cells. Triangles mark the nuclei of the APC. The macrophage incubated with α-gal presenting lymphoma cells internalized nine cells, and the dendritic cell internalized one α-gal presenting lymphoma cell. No uptake of lymphoma cells lacking α-gal epitopes was observed (×1,000). Adapted with permission from Manches et al. (2005).

The effective uptake by macrophages and dendritic cells of particulate anti-Gal/α-gal epitope immune complexes is further presented in Figure 7, which describes uptake by APC of freshly obtained human lymphoma cells opsonized by anti-Gal. As described in the section on anti-Gal-mediated conversion of human tumors into autologous anti-tumor vaccines, *in situ* immunocomplexing of anti-Gal with tumor cells presenting α-gal epitopes can result in effective targeting of the tumor cells to APC due to the Fc tail of anti-Gal binding to Fc receptors on macrophages and dendritic cells. The subsequent transport to regional lymph nodes, processing, and presentation of autologous tumor antigens by the APC can elicit a protective immune response against metastatic tumor cells. To demonstrate this uptake, human lymphoma cells were glycoengineered to present α-gal epitopes by a two-step enzymatic reaction (Figure 7A), in which the sialic acid was removed from carbohydrate chains of cell surface glycoproteins and glycolipids by neuraminidase. Subsequently, the α-gal epitope was synthesized on these carbohydrate chains by recombinant α1,3-galactosyltransferase (α1,3-GT), which links galactose α1-3, provided by UDP-Gal, to the carbohydrate chains, resulting in the formation of >10^6^ α-gal epitopes per cell (LaTemple et al., 1996). Incubation at 37°C for 2 h of lymphoma cells presenting α-gal epitopes with the patient’s macrophages in the presence of autologous anti-Gal resulted in extensive uptake of the tumor cells by the macrophages, whereas the original tumor cells lacking α-gal epitopes were not phagocytosed by the macrophages (Figure 7B) (Manches et al., 2005). Similarly, dendritic cells internalized the anti-Gal-coated lymphoma cell, whereas no such uptake was observed with the original tumor cells lacking anti-Gal epitopes. As summarized in the following section, α-gal epitopes can be expressed on viruses, tumor cells, and nanoparticles. The interaction of anti-Gal with particulates presenting α-gal epitopes can induce the amplification of viral and autologous tumor vaccines and induce accelerated healing and regeneration in various clinical settings.

### Increased efficacy of vaccines against enveloped viruses

The recent experience with gene-based COVID-19 vaccines indicated that although such vaccines provide protection against the virus, they do not prevent the appearance of variants that contain mutations in the S-protein gene. These mutations enable the variant virus to escape the anti-S protein antibodies in vaccinated individuals. One method to prevent the appearance of such variants is the use of inactivated whole virus vaccine internalized by APC such as dendritic cells and macrophages. The APC transport the vaccinating virus to the regional lymph nodes and process and present viral peptides for the induction of a protective immune response against multiple viral antigens. Thus, if the virus acquires escape mutations in the S-protein, the immune response against other viral antigens will result in the destruction of the mutated virions before they expand into new variants. However, in enveloped virus vaccines, including SARS-CoV-2 causing COVID-19, the uptake of the whole virus vaccine by APC may be suboptimal because of the multiple carbohydrate chains on envelope glycoproteins, which form the “glycan shield” that masks viral antigens. The glycan shield further presents negative charges that electrostatically deflect the vaccinating virions from the APC membranes. This deflection is mediated by multiple negatively charged sialic acid units on both the virus carbohydrate chains and those on the APC membrane glycoproteins; both are similar to the left carbohydrate chain in Figure 7A (Galili, 2020).

**FIGURE 8:**
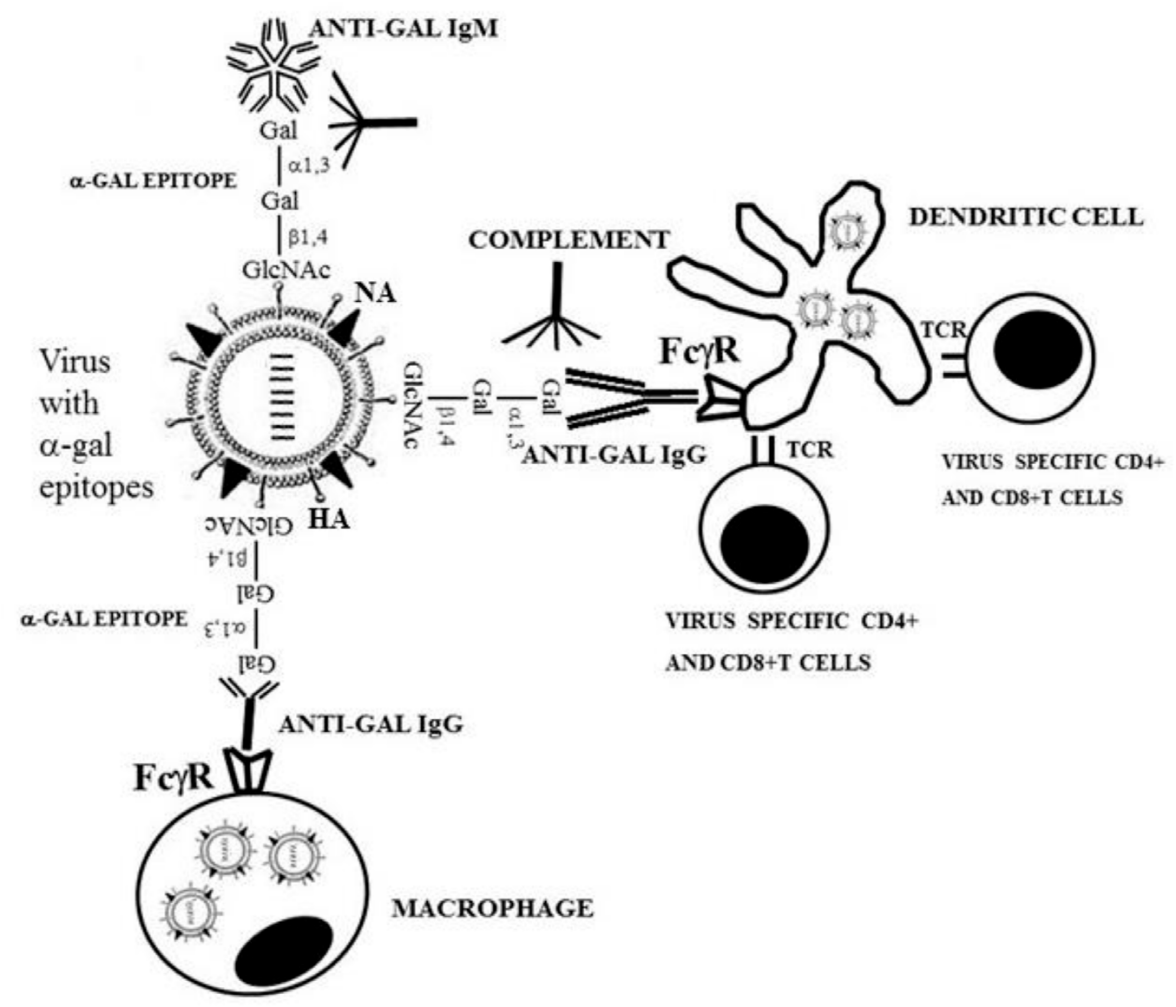
Amplification of viral vaccine immunogenicity by anti-Gal-mediated targeting of the vaccinating virus to APC. Influenza virus glycoengineered to present α-gal epitopes is used as an illustrative example for α-gal inactivated whole virus vaccine. Anti-Gal IgM and IgG bind at the vaccination site to α-gal epitopes on the vaccinating virus. This anti-Gal/α-gal epitope interaction activates the complement system, resulting in the release of complement cleavage chemotactic peptides C5a and C3a that recruit APC, such as dendritic cells and macrophages, to the vaccination site. Anti-Gal IgG coating the virus mediates its extensive uptake by the recruited APC via Fc/Fcγ receptors (FcγR) interaction. C3b/C3b receptor interaction on APC also may contribute to the extensive uptake of the virus vaccine. APC transport the internalized virus vaccine to the regional lymph nodes and process and present the viral immunogenic peptides on class I and class II MHC molecules for the activation of virus-specific CD8^+^ and CD4^+^ T cells, respectively. HA, hemagglutinin; NA, neuraminidase; TCR, T-cell receptor. Reproduced from Galili U. book “The natural anti-Gal antibody as foe turned friend in medicine,” Academic Press/Elsevier, London, 2018, with permission.

The uptake by APC of whole virus vaccines can be markedly increased by converting the terminal sialic acid on viral carbohydrate chains into α-gal epitopes by enzymatic removal of the sialic acid with neuraminidase and linking terminal α1,3-galactose by recombinant α1,3-galactosyltransferase for the formation of α-gal epitopes, as shown in Figure 7A (Galili, 2020; Galili, 2021). Immunization with viral vaccines presenting α-gal epitopes results in the binding of anti-Gal IgG to these epitopes and the activation of the complement system. The complement cleavage peptides C5a and C3a are potent in inducing chemotaxis of APC to the vaccination site (Figure 8). The Fc “tail” of anti-Gal IgG bound to the α-gal epitopes on the glycoengineered vaccinating virus binds to Fc receptors on APC and induces extensive uptake of the vaccinating virions. This, in turn, results in effective transport, processing, and presentation of multiple vaccinating virions, which induce an immune response that is much higher than that measured with the unmodified inactivated virus (Figure 8). Studies with influenza virus vaccine glycoengineered to present α-gal epitopes demonstrated a ∼100-fold increase in anti-viral antibody production and ∼9-fold increase in protection against infection with a lethal dose of the virus compared to mice immunized with the unmodified viral vaccine (Abdel-Motal et al., 2007). Thus, it is possible that vaccination of humans with inactivated SARS-CoV-2 virus or other enveloped virus vaccines glycoengineered to present α-gal epitopes will be much more effective in inducing a protective immune response against several viral antigens than virus vaccines with unmodified carbohydrate chains. In addition to the use of recombinant α1,3-galactosyltransferase for glycoengineering of viral vaccines, propagating the vaccinating virus in host cells transduced with AdαGT or propagated in cells stably transfected with several copies of the α1,3-GT gene *GGTA1* may result in the production of virions with multiple α-gal epitopes (Galili, 2020; Galili, 2021).

### The double-edge sword of α-gal epitopes on therapeutic recombinant glycoproteins

The presentation of α-gal epitopes on therapeutic recombinant glycoproteins depends on the activity of α1,3-GT in the cells producing the recombinant protein. Recombinant human interferon-β1 was produced by Chinese hamster ovary (CHO) cells, mouse epithelial cells (C127), and human lung adenocarcinoma cells (PC8) and analyzed for α-gal epitope expression (Kagawa et al., 1988). Whereas the α1,3-GT negative cells CHO and PC8 produced recombinant human interferon-β1 lacking α-gal epitopes, the α1,3-GT-positive C127 cells produced recombinant human interferon-β1 that presented several α-gal epitopes on its N-linked carbohydrate chains (Kagawa et al., 1988). The reasons for the absence of α1,3-GT in the hamster cells CHO are discussed by Galili (2018). The presence of α-gal epitopes on therapeutic glycoproteins may have clinical implications for the therapeutic efficacy of such glycoproteins. The binding of anti-Gal to the α-gal epitopes following administration of such glycoproteins into humans may result in the formation of immune complexes that are removed by the reticuloendothelial system in a faster manner than non-immunocomplexed recombinant glycoproteins. This was demonstrated with monoclonal antibodies produced in hybridoma cells containing or lacking α1,3-GT (Borrebaeck et al., 1993). The antibodies were intravenously introduced to the patients, and the half-life of these antibodies in the circulation was determined. Whereas monoclonal antibodies lacking α-gal epitopes had a half-life of at least a week, the half-life of monoclonal antibodies presenting α-gal epitopes was found to be only 19–43 h. The decrease in half-life was proportional to the number of α-gal epitopes on each of these antibodies (Borrebaeck et al., 1993). In contrast to the negative effects of α-gal epitopes on the half-life of therapeutic recombinant glycoproteins in circulation, α-gal epitopes on recombinant glycoproteins used as vaccines markedly increase the immunogenicity of such vaccines. This increase was mediated by a mechanism similar to that illustrated in Figure 8 for whole virus vaccines presenting α-gal epitopes. Immunization of anti-Gal producing GT-KO mice with HIV recombinant gp120-presenting α-gal epitopes on the multiple carbohydrate chains of this recombinant protein resulted in a ∼100-fold increase in antibody production against the HIV envelope glycoprotein gp120, compared to the same vaccine lacking α-gal epitopes (Abdel-Motal et al., 2006). This was due to the vigorous targeting of the immunocomplexed gp120 vaccine to APC at the vaccination site (Abdel-Motal et al., 2006). Similarly, a 30-fold increase in antibody production to gp120 and p24 was observed in GT-KO mice immunized with a fusion protein of gp120 and p24 (an internal HIV protein lacking carbohydrate chains) that presents α-gal epitopes only on the gp120 portion of the recombinant fusion glycoprotein, compared to the same protein lacking α-gal epitopes (Abdel-Motal et al., 2010). Thus, the presentation of multiple α-gal epitopes on recombinant glycoprotein vaccines may result in a marked increase in the efficacy of such vaccines.

### *In situ* conversion of tumors into autologous tumor vaccines

All cancers arise as a result of somatically acquired changes in the DNA of cancer cells. These mutations can be either “driver” mutations associated with oncogenesis or “passenger” mutations that do not contribute to cancer development (Stratton et al., 2009). Regardless of their contribution to cancer development, if both types of mutations result in changes in amino acid sequence in cellular proteins, some of the mutated proteins may be “regarded” by the immune system as foreign antigens that elicit a protective immune response. This assumption is supported by observations reporting a correlation between the extent of T-cell infiltration into primary or metastatic tumors, a decrease in growth of the primary tumor and metastatic spread, and improved clinical outcome (Zhang et al., 2003; Galon et al., 2006; Mlecnik et al., 2011). In patients with low or no infiltration of T cells and, accordingly, with poor prognosis, it is probable that the immune system fails to detect the mutated proteins and react against them. Based on these considerations, it was argued that tumors might be converted into *in situ* vaccines against the mutated proteins in the individual patient and that such conversion is feasible by inducing the expression of α-gal epitopes on tumor cell membranes (Galili and LaTemple, 1997). As in the aforementioned case of glycoengineered viral vaccines for expression of α-gal epitopes (Figure 8), it was assumed that binding of the patient’s anti-Gal to autologous tumor cells glycoengineered to present α-gal epitopes might result in complement activation, recruitment of APC that will effectively phagocytose the anti-Gal opsonized tumor cells, and cell membranes (Figure 7B). APC process and transport the mutated tumor cell peptides to regional lymph nodes, resulting in the induction of a protective immune response against the treated tumor and metastatic cells. This protective immune response may be highly variable and will depend on the immunogenicity of the mutated tumor cell proteins, as well as the activity of the immune system in the treated patient. However, the treatment is customized to the mutated proteins of the individual patient, and it does not require the identification of these mutations.

Experimental studies with the mouse B16 melanoma cell lines (cells lack α-gal epitopes) in anti-Gal-producing GT-KO mice were performed to study the hypothesis on the increased immunogenicity of tumors engineered to present α-gal epitopes. B16 cells stably transfected with the α1,3-GT gene *GGTA1* for the induction of α-gal epitopes presentation were used as a vaccine. This vaccine was found to be much more effective in inducing a protective immune response against challenges with live B16 cells and against the development of distant metastases than unmodified B16 vaccinating cells (LaTemple et al., 1999; Rossi et al., 2005). A practical and effective way for *in situ* conversion of a solid tumor into an autologous tumor vaccine presenting α-gal epitopes was the intra-tumoral injection of natural α-gal glycolipid micelles (Galili et al., 2007; Abdel-Motal et al., 2009) or such synthetic α-gal glycolipid micelles (Shaw et al., 2019). Intra-tumoral injections of both natural and synthetic α-gal glycolipid micelles were found to decrease the size of the treated tumor and prevent the development of distant metastases (Galili et al., 2007; Abdel-Motal et al., 2009; Shaw et al., 2019). Intra-tumoral injection of α-gal glycolipids in a small group of cancer patients in an advanced state of the disease was found in a Phase-1 clinical trial to be well-tolerated with no toxic effects (Whalen et al., 2012).

### Acceleration of wound and burn healing and prevention of scar formation

The physiologic repair mechanism of skin wounds in adult mammals and humans involves the migration of macrophages to the wound, followed by debriding of the wound by these macrophages (called “pro-inflammatory” macrophages). Subsequently, pro-reparative macrophages mediate the repair by fibrosis of the wound and scar formation at the site of the healed wound (Singer and Clark, 1999; Gunter et al., 2008). The healing of wounds in amphibian urodeles (newt, salamander, and axolotl) also involves the migration of macrophages into the injury site. However, the healing mediated by these macrophages (called “pro-regenerative macrophages”) results in the complete regeneration of the normal structure and function of the skin, as that prior to the injury (Lévesque et al., 2010; Mastellos et al., 2013; Godwin and Rosenthal, 2014; Natarajan et al., 2018). One of the main characteristics differentiating between wound healing by scar formation in adult mammals and wound regeneration in urodeles is the involvement of the complement system activated by non-immune mechanisms in the latter amphibian group (Mastellos et al., 2013; Natarajan et al., 2018).

**FIGURE 9:**
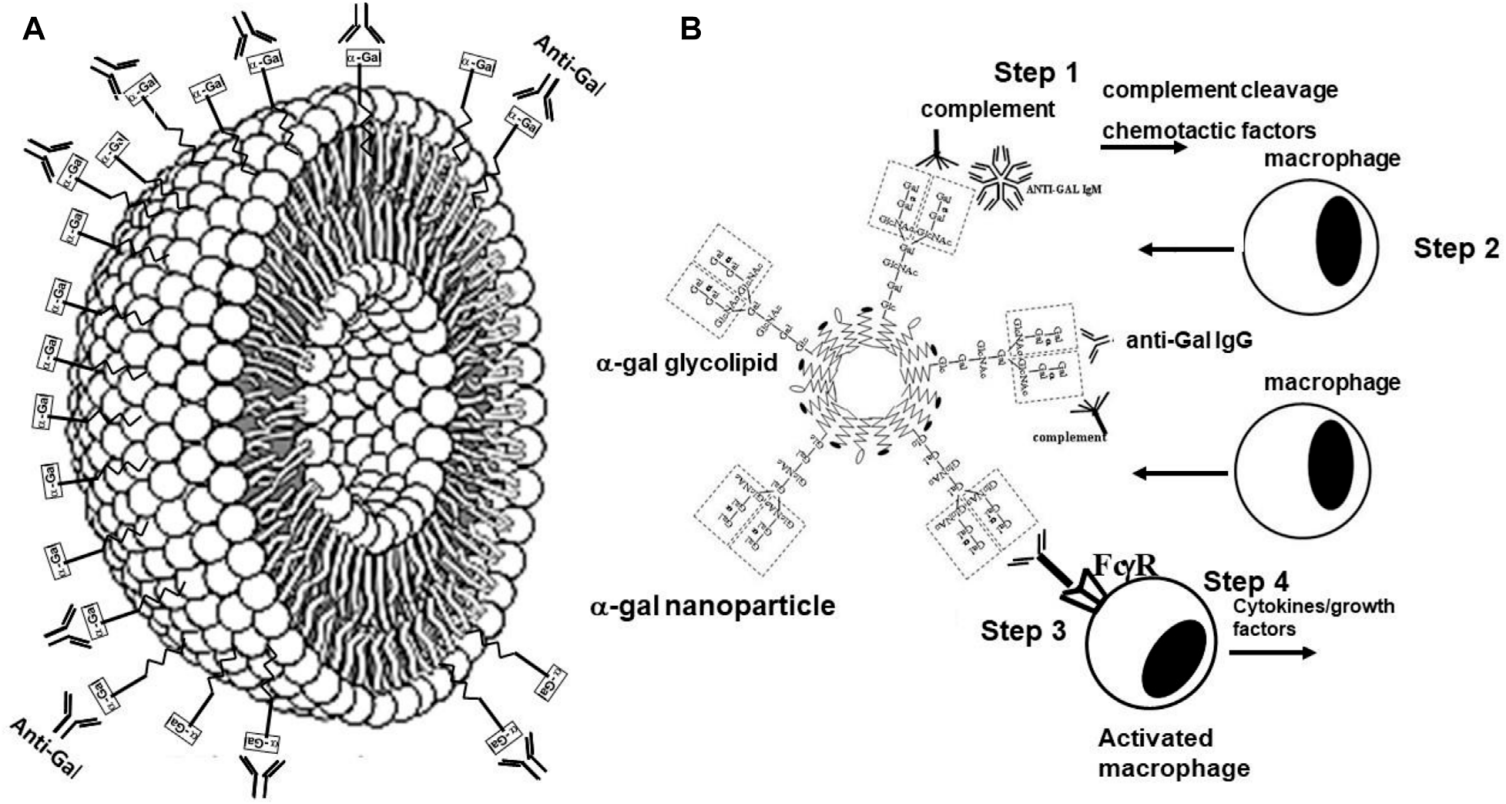
Mechanism for the regenerative effects of α-gal nanoparticles in injuries. **(A)** Schematic section in α-gal nanoparticles illustrating the phospholipids forming a lipid bilayer of the nanoparticle wall, in which multiple glycolipids with α-gal epitopes (rectangles) are anchored. Upon administration into various injured tissues, the anti-Gal antibody, which is abundant in the serum, readily binds to the α-gal epitopes on the α-gal nanoparticles. **(B)** Steps in the activity of α-gal nanoparticles administered to injuries. 1) Binding of natural anti-Gal to α-gal nanoparticles activates the complement system and results in the formation of the chemotactic complement cleavage peptides C5a and C3a. 2). The chemotactic factors C5a and C3a induce extensive recruitment of macrophages to the site of α-gal nanoparticles. 3) The recruited macrophages bind via their Fcγ receptors (FcγR) the Fc portion of anti-Gal coating the α-gal nanoparticles. 4) This interaction induces polarization of the cells into pro-regenerative macrophages that secrete a wide range of cytokines and growth factors, which accelerate the healing of the treated injuries and prevent scar formation. Reproduced from Galili U. book “The natural anti-Gal antibody as foe turned friend in medicine.” Academic Press/Elsevier, London, 2018, with permission.

Based on the aforementioned considerations, it was hypothesized that localized complement activation in adult-mouse injuries may induce skin regeneration, as in urodeles, instead of healing by the default process of fibrosis and scar formation. In view of the extensive ability of the natural anti-Gal antibody to activate the complement system following binding to α-gal epitopes described previously, it was further hypothesized that localized complement activation within wounds might be achieved by the administration of biodegradable nanoparticles presenting multiple α-gal epitopes (Galili et al., 2010; Wigglesworth et al., 2011; Galili, 2017; Kaymakcalan et al., 2018). Such complement activation is feasible by antigen/antibody interaction between biodegradable nanoparticles presenting α-gal epitopes (called α-gal nanoparticles) and the natural anti-Gal antibody abundant in humans. Biodegradable α-gal nanoparticles were generated from a mixture of glycolipids, phospholipids, and cholesterol extracted from rabbit red cell membranes, which are rich in α-gal glycolipids (Figure 9A) (Galili et al., 1987a). In an analogy with the administration of α-gal virus vaccines, the application of α-gal nanoparticles on wounds is expected to result in the following steps (Figure 9B): Step 1: The natural anti-Gal antibody binds to α-gal nanoparticles administered to the wound and activates the complement system that produces complement cleavage chemotactic peptides C5a and C3a. Step 2: C5a and C3a direct the recruitment of macrophages to the injury site. Step 3: The Fc portion of anti-Gal bound to α-gal nanoparticles binds to Fcγ receptors (FcγR) of recruited macrophages, resulting in their activation into pro-regenerative macrophages. A similar binding may occur between C3b on the α-gal nanoparticles and C3b receptors on macrophages. Step 4: The activated macrophages induce the regeneration of the injured tissue by secreting pro-regenerative cytokines/growth factors.

Studies on α-gal nanoparticles wound treatment in anti-Gal-producing GT-KO mice have demonstrated a decrease in the healing time of full-thickness wounds from 12–14 days to 6 days. When the wounds were inspected histologically after 28 days, control wounds treated with saline displayed distinct fibrosis and scar formation, including hypertrophic epidermis, dense connective tissue, and absence of skin appendages. In contrast, wounds treated with α-gal nanoparticles displayed the regeneration of injured skin, characterized by the normal thin epidermis, loose connective tissue, and appearance of skin appendages, including hair shafts, sebaceous glands, smooth muscle cells, and adipocytes (Wigglesworth et al., 2011; Galili, 2017). The prevention of scar formation in skin wounds may be associated with the accelerated healing induced by α-gal nanoparticles, which occurs prior to the “kicking in” of the default repair mechanism of fibrosis and scar formation. Similar accelerated healing by α-gal nanoparticles treatment was observed in GT-KO pigs producing the natural anti-Gal antibody (Hurwitz et al., 2012). Accelerated healing with kinetics similar to that in wounds was also observed in anti-Gal-producing GT-KO mice with thermal skin injuries (Galili et al., 2010; Samadi et al., 2022). The ability of α-gal nanoparticles to induce wound regeneration was further found to induce the healing of chronic wounds in mice with chemically induced diabetes (Galili, 2017; Kaymakcalan et al., 2020). All these studies suggest that α-gal nanoparticles injury treatment may be an appropriate candidate for studying accelerated healing and the regeneration of external and internal injuries in humans.

### Induction of myocardium regeneration following myocardial infarction

Healing of the heart muscle (myocardium) in adult mammals following myocardial infarction (MI) results in fibrosis and scar formation. Although this healing process reduces in humans the risk of death from spontaneous rupture of the myocardium wall, the absence of any myocardial regeneration results in reduced contractility, adverse ventricular remodeling, and left ventricle dilation, which can lead to congestive heart failure and premature death. The post-MI myocardial repair mechanism involves macrophages similar to the process of wound healing in mammals described previously (Nahrendorf et al., 2007; Frangogiannis, 2018; Lavine et al., 2018). In contrast, injured myocardium in amphibians, such as axolotl and salamanders, displays spontaneous regeneration, which involves non-immune activation of the complement system (Natarajan et al., 2018) and macrophage migration into the injured myocardium (Godwin et al., 2017). These observations raised the question of whether α-gal nanoparticles can induce the regeneration of the injured myocardium after MI in adult mice. This was studied in anti-Gal-producing GT-KO mice, in which MI was induced by occluding the mid-portion of the left anterior descending (LAD) coronary artery by ligation with a silk suture for 30 min. Subsequently, the occlusion was removed, and the re-perfused injured myocardium received two 10 µL injections of 10 mg/mL α-gal nanoparticles or two 10 µL injections of saline as control (Galili et al., 2021). Planimetry measurements of the extent of fibrosis and scar formation were performed in the nanoparticle- and saline-treated mice 28 days after MI. These measurements demonstrated in control mice large transmural infarcts with extensive scar formation in 20%–30% of the left ventricular wall. Echocardiography studies demonstrated poor contractile function 7 and 28 days after MI. In contrast, fibrosis and scar formation in the hearts of mice treated with α-gal nanoparticles were minimal, the infarct size was ∼10-fold smaller than that in control saline-treated mice, and the ventricular wall displayed restoration of normal myocardium structure. In addition, echocardiography studies demonstrated in these mice poor contractility 7 days after MI but restoration of normal myocardium contractile function 28 days after MI (Galili et al., 2021). In contrast, in saline-treated mice, the poor contractile function observed 7 days after MI was also observed 28 days after MI, implying permanent damage to the myocardium. Overall, these findings demonstrate near complete restoration of normal structure and function in post-MI adult mice treated with α-gal nanoparticles, similar to the physiologic restoration of normal structure in injured hearts of salamander and axolotl and similar to wounds treated with these nanoparticles.

## Conclusion

The understanding of the immune response in humans against incompatible blood-group A and B antigens on allografts is limited to the results observed in recipients of such grafts. The results point to the following mechanisms of the immune response: 1) hyperacute or chronic rejection of the A or B incompatible graft by natural or elicited anti-A or anti-B antibodies. 2) In patients in whom T-cell activity is suppressed by immunosuppressive drugs, anti-A or anti-B antibodies are removed, and the size of the immune system is decreased by splenectomy or by immune-mediated destruction of B cells, the immune response may result in the production of accommodating anti-A or anti-B antibodies. These antibodies bind to the incompatible A or B antigen on the endothelial cells of the graft but do not activate the complement system and therefore do not damage the endothelial cells, thus enabling the survival of the graft for prolonged periods. 3) In some of the treated patients, immune-tolerance induction to the incompatible A or B antigen results in the absence of anti-A or anti-B antibodies and, thus, the long-term survival of the allograft.

**FIGURE 10:**
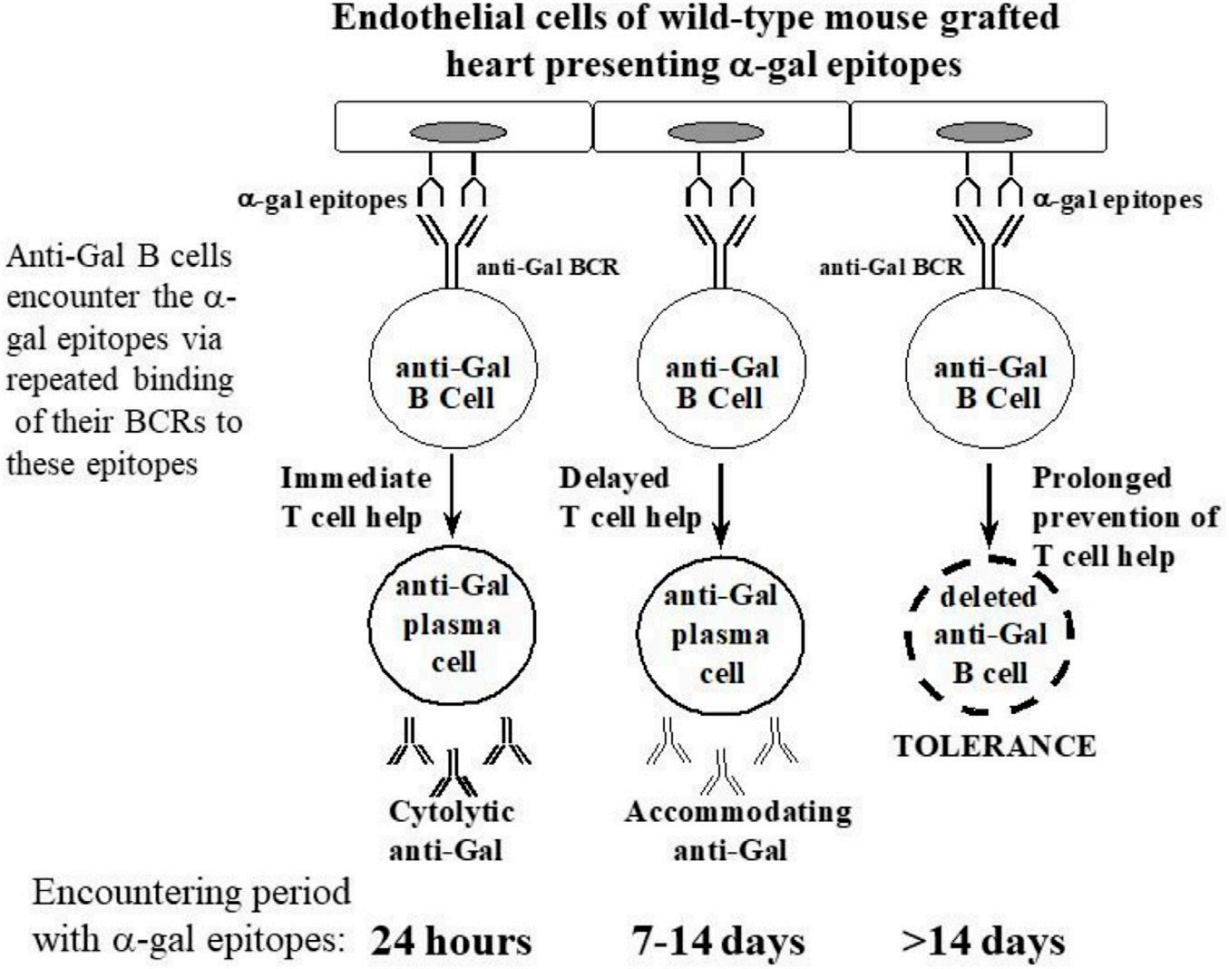
The effects of repeated encounters between anti-Gal B cells and α-gal epitopes on endothelial cells of syngeneic wild-type (WT) heart grafts in GT-KO mice, in the absence of T-cell help, on the production of anti-Gal. When T-cell help is provided within 24 h by immunization with PKM, exposure to α-gal epitopes activates anti-Gal B cells into plasma cells producing cytolytic anti-Gal antibodies. If T-cell help is provided to anti-Gal B cells only after 7–14 days of repeated encounters with α-gal epitopes, these B cells differentiate into plasma cells producing accommodating anti-Gal antibodies. Repeated encounters of naïve or memory anti-Gal B cells for >14 days, in the absence of T-cell help, result in tolerance to the α-gal epitope due to the elimination of anti-Gal B cells either by deletion or by Ig receptor editing. BCR, B-cell receptor. Modified from Galili U. book “The natural anti-Gal antibody as foe turned friend in medicine.” Academic Press/Elsevier, London, 2018” with permission and based on Galili (2004).

Immune response to the α-gal epitope, which results in the production of the natural anti-Gal antibody in GT-KO mice immunized with pig kidney membranes (PKM), was chosen as the experimental model for a better understanding of the principles underlying the different types of the immune response against incompatible A or B antigen in human allografts. This system was chosen because the α-gal epitope is the core of blood-group A and B antigens and because anti-Gal comprises >85% of anti-blood-group B antibody activity in A and O individuals. In GT-KO mice producing anti-Gal, grafting of heterotopic syngeneic WT mouse hearts presenting α-gal epitopes can result in three types of immune response (Figure 10) (Galili, 2004). 1) In anti-Gal-producing mice, grafted WT heart results in hyperacute rejection as in humans. 2) If anti-Gal B cells in recipients of WT heart repeatedly encounter for ∼7 days the α-gal epitope on the heart graft, in the absence of T-cell help, these B cells undergo isotype switch, which results in the extensive production of anti-Gal IgG2b antibody that functions as an accommodating antibody which prevents WT heart rejection. 3) Repeated encounters of anti-Gal B cells for 14 days or more with the α-gal epitope on the heart graft and in the absence of T-cell help cause the elimination of these B cells by either apoptosis or Ig receptor editing. This results in the induction of immune tolerance to the α-gal epitope and long-term survival of the WT heart graft. This survival is maintained despite repeated immunization with PKM (which elicits anti-Gal production in mice that are not tolerized). This tolerance is induced in both naïve and memory anti-Gal B cells. A similar tolerance to the α-gal epitope can also be induced within 14 days, in the absence of T-cell help, by the administration of syngeneic WT lymphocytes or GT-KO lymphocytes glycoengineered to present α-gal epitopes. Such glycoengineering is achieved by the transduction of GT-KO lymphocytes with replication-defective adenovirus containing the α1,3-galactosyltransferase gene. It is suggested that a similar tolerance induction to incompatible A and B antigens may be feasible in patients receiving autologous lymphocytes engineered to present these incompatible carbohydrate antigens prior to the transplantation of the allograft.

As the natural anti-Gal antibody is active in all humans, it can be exploited for beneficial therapeutic effects in various clinical settings, including: 1) Glycoengineering of inactivated whole virus vaccines to present α-gal epitopes may greatly increase the efficacy of such vaccines. This increased efficacy is achieved due to the extensive anti-Gal mediated targeting of the vaccinating virus to antigen-presenting cells (APC). 2) Administration of α-gal glycolipids into solid tumors results in *in situ* presentation of α-gal epitopes on tumor cells. This leads to a marked increase in anti-Gal-mediated uptake of tumor cells and cell membranes by APC and conversion of treated tumors into autologous tumor vaccines. Such vaccines elicit a protective immune response against autologous tumor antigens in the individual patient, thereby inducing immune-mediated destruction of the treated tumor and distant metastases. 3) Administration of α-gal nanoparticles into external (e.g., skin wounds) or internally injured tissues (e.g., post-MI injured myocardium) results in anti-Gal-mediated recruitment of pro-regenerative macrophages that mediate the regeneration of the structure and function of the injured tissue, thereby avoiding scar formation.
